# Whole blood transcriptomics atlas across human diseases

**DOI:** 10.1038/s41467-026-78440-w

**Published:** 2026-10-12

**Authors:** Mengzhen Li, Abdulgani Tatar, Emre Green, Ebru Simsek, Cigdem Yuce Kahraman, Mustafa Ceylan, Fuat Laloglu, Naci Ceviz, Arzu Tatar, Esra Laloglu, Gungor Akcay, Kenan Cadirci, Omer Erkan Yapca, Hilal Kiziltunc Ozmen, Turgut Yapanoglu, Ozlem Kudas, Fatma Simsek, Emsal Pinar Topdagi Yılmaz, Gamze Nur Cimilli Senocak, Hakan Doneray, Ayse Ozden, Atilla Cayir, Pinar Tosun Tasar, Mustafa Kara, Yunus Emre Aktaş, Mursel Karadavut, Busra Akpinar, Eyup Serhat Calik, Bilgehan Erkut, Mufide Akcay, Erdem Karadeniz, Murat Kartal, Esra Disci, Mehmet Ilhan Yildirgan, Sabri Selcuk Atamanalp, Rifat Peksoz, Yavuz Albayrak, Atilla Eroglu, Yener Aydın, Sultan Tuna Akgol Gur, Mevlana Gul, Erdal Tekin, Ali Bilal Ulas, Eyup Senocak, Ahmet Emre Paksoy, Ayse Sena Donmez, Mehmet Cenk Turgut, Ozgur Ozmen, Fatih Alper, Hasan Abbas Guliyev, Bulent Albayrak, Emine Cinici, Ibrahim Kocer, Esat Fahri Aydın, Nazim Dogan, Yahya Gul, Ugur Kaya, Yunus Emre Topdagi, Yakup Kumtepe, Aysenur Dostbil, Husnu Kursat, Nuray Bilge, Ozkan Cinici, Esra Disci, Derya Cevizli, Muhammed Akif Guler, Halil Ozcan, Tugba Koca Lacin, Serap Sari, Bugra Kerget, Omer Araz, Fatma Tortum, Necip Altundas, Nurhak Aksungur, Salih Kara, Gurkan Ozturk, Serap Kilic Kaya, Merve Ulku, Fatma Kesmez Can, Handan Alay, Ayse Albayrak, Kemalettin Ozden, Ertugrul Karatas, Mirze Muhammet Ozen, Ali Gur, Taner Arabaci, Sinan Yilar, Serdar Saritas, Zulal Ozkurt, Hacer Akgul Ceyhun, Can Sevinc, Mehmet Akif Sirin, Gulden Sincan, Momen Kanjee, Busra Saruhan, Sumeyye Ozturk Eren, Bahadir Utlu, Muhammed Hakan Tas, Esra Tanriverdi, María Bueno Álvez, Fredrik Edfors, Xiangtai Kong, Xiya Song, Ozlem Altay, Bayram Yuksel, Mathias Uhlen, Saeed Shoaie, Cheng Zhang, Hasan Turkez, Adil Mardinoglu

**Affiliations:** 1 Science for Life Laboratory, KTH – Royal Institute of Technology, Stockholm, Sweden; 2 Faculty of Medicine, Atatürk University, Erzurum, Turkey; 3 SZA Omics R&D, Acibadem Mehmet Ali Aydinlar University, Istanbul, Turkey; 4 Erzurum Regional Training and Research Hospital Health Sciences University, Erzurum, Turkey; 5 Faculty of Dentistry, Atatürk University, Erzurum, Turkey; 6 Centre for Host-Microbiome Interactions, Faculty of Dentistry, Oral & Craniofacial Sciences, King’s College London, London, UK; 7 Roger Williams Institute of Liver Studies, School of Immunology & Microbial Sciences, Faculty of Life Sciences and Medicine, King’s College London, Foundation for Liver Research, and King’s College Hospital, London, UK

**Keywords:** High-throughput screening, Predictive markers

## Abstract

We present a WBT Atlas, comprising 4444 WBT samples across 98 distinct health conditions, enabling systematic exploration of disease-associated transcriptomic signatures. Health condition–specific profiles are characterized across immune, metabolic, and cell-type specific features at the gene, gene module, and pathway levels. By integrating matched plasma proteomics data from 1280 participants across 28 health conditions, we further assess blood RNA–protein relationships and demonstrate their complementary biological roles. By integrating RNA and protein signatures, we develop multi-class classification models that accurately distinguish among a wide range of health conditions, with integrated models consistently outperforming those based on single-omics data alone. The identified RNA and protein signatures are validated across ten independent WBT cohorts and the UK Biobank. Together, the WBT Atlas provides a genome-wide resource that enables translational discovery in precision medicine.

## Abstract (web summary)

The Whole Blood Transcriptome database comprises data from 4,444 individuals with 98 distinct health conditions and matched plasma proteomics data from 1,280 with 28 health conditions, allowing identification of signatures of the conditions.

## Introduction

The human population exhibits high genetic and phenotypic diversity, shaped by factors such as age, sex, and chronic diseases^1^. Systematic understanding of the molecular signatures that reflect this diversity, particularly across different health conditions, requires the construction of large-scale and well-curated biological data resources. In recent years, landmark initiatives such as The Human Protein Atlas (HPA)^2^, UK biobank^3^, The Cancer Genome Atlas (TCGA)^4^, and Genotype-Tissue Expression (GTEx)^5^ have provided foundational platforms for mapping the molecular landscapes of human cells and tissues across health and disease. By capturing biological heterogeneity at the population scale, these resources have enabled the identification of key molecular signatures and biological pathways with diagnostic and therapeutic relevance^6^.

Building on these efforts, several large-scale plasma proteomics studies have demonstrated the power of blood-based molecular profiling for disease characterization. For instance, Deng et al^7^. constructed a proteomics atlas comprising 2920 plasma proteins associated with diverse diseases and developed protein-based predictive models for disease prediction. Similarly, Álvez et al. developed a pan-cancer classification model based on plasma proteomics profiles comprising 1463 proteins across 12 cancer types^8^, and later expanded the analysis to 8312 individuals spanning 59 diseases^9^. These studies underscore the potential of circulating biomarkers for non-invasive disease diagnosis and monitoring.

While plasma proteomics provides essential insights, transcriptomics represents a comprehensive layer of molecular information. The cellular phenotype is primarily governed by its transcriptomics landscape^10^, which typically encompasses the expression profiles of nearly 20,000 protein-coding genes in humans. Whole blood transcriptomics (WBT) provides a broader and more detailed view of biological processes, primarily in immune cells of the blood, and is therefore an attractive complement to analyses of the circulating blood proteome and to conventional immune cell analyses using flow sorting. Moreover, WBT profiles have demonstrated classification performance comparable to tissue-derived transcriptomics^11^, with the added advantages of accessibility and the ability to capture systemic immune and metabolic states. Despite these advantages, a comprehensive, disease-focused WBT atlas remains lacking.

To address this gap, we present a large-scale WBT Atlas comprising 4444 individuals across 98 distinct health conditions (https://www.phenomeatlas.org/wbt/). Through integrative analyses, we systematically characterized both demographic and disease-associated transcriptomics patterns, including aging, immune, metabolic, and cell–type specific features. In addition, matched plasma proteomics data from 1280 participants across 28 health conditions enabled RNA–protein correlation analyses, revealing complementary features between WBT and plasma proteomics. We further identified disease-specific transcriptomics and proteomics signatures and developed multi-class disease classification models. The selected features were subsequently validated in ten independent WBT cohorts and the UK Biobank, comprising 1621 and 9998 samples, respectively. Together, this work provides a comprehensive resource for exploring WBT signatures across a broad spectrum of health conditions and demonstrates their complementarity to proteomics.

## Results

### Disease shapes blood transcriptomes across lifespan

**Fig. 1:**
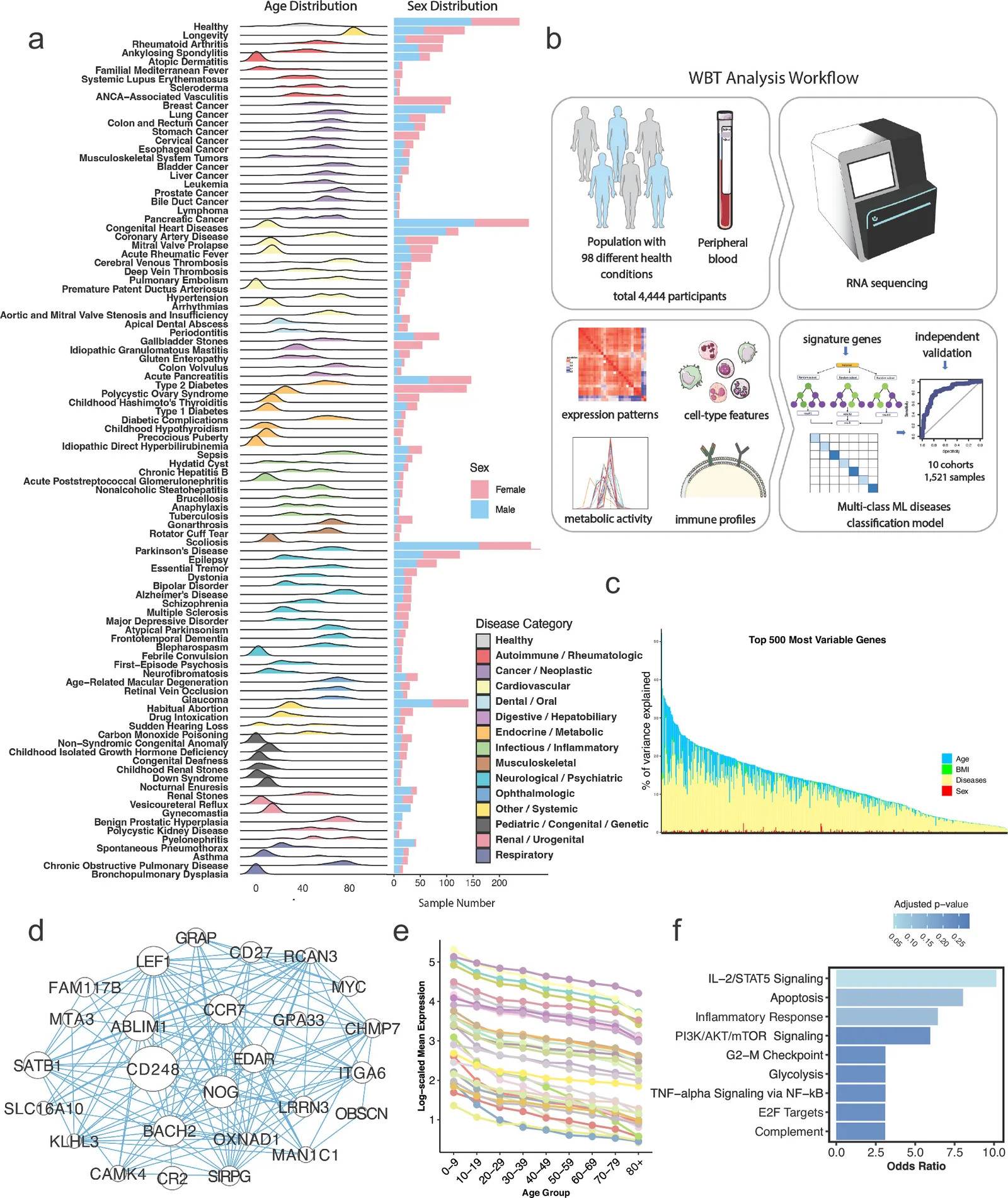
Demographic-associated whole blood transcriptomic signatures. **a** Age, sex distribution and sample counts across health conditions included in the study. **b** Workflow of whole blood transcriptomic data analysis. **c** Proportion of variance explained by sex, age, and health conditions for the top 500 most variable genes across 4444 participants. **d** Aging associated gene cluster comprising 32 genes are shown as a correlation network. Each node represents a gene, with node size indicating the η² effect size (variance explained by age), and edge width reflecting the strength of pairwise expression correlations. **e** Expression trends of aging associated genes across age decades, showing a consistent pattern of age-associated downregulation. **f** Enriched MSigDB pathways identified from the 32 aging associated genes. Source data are provided in the Source data file.

To systematically analyze WBT features, we collected peripheral blood samples from 4444 participants with 98 distinct health conditions (Fig.1b). Detailed information on sample size, sex, and age distribution for each condition is provided in Supplementary Table 1. Overall, participants in this cohort ranged in age from 0 to 99 years, with a mean age of 42.3 ± 25.1 years. The sex distribution was 48.7% male and 51.3% female. The 98 included health conditions were categorized into 15 broader groups based on disease mechanisms or pathological locations (Fig.1a). We first examined the factors that contribute to gene expression variance. To achieve this, we modeled gene abundance using age, sex, BMI, and health conditions through ANOVA and quantified the proportion of variance explained by each factor using Eta-squared (η²). As shown in Fig. 1c, health condition accounted for the largest proportion of variance across most genes, highlighting its dominant influence on WBT profiles.

As age explained the second-highest proportion of variance, we also explored aging-associated gene signatures in WBT profiles. To identify aging-associated genes, we focused on those for which age accounted for the most significant proportion of expression variance (η² from ANOVA). Notably, these top genes showed strong concordance with the aging-related transcriptomics signatures that have been previously reported^12^. The overlap proportion ranged from 80% for the top 10 genes to 61% for the top 100 genes (Supplementary Fig. 1a). We subsequently selected the 32 genes that overlapped within the top 50 candidates (η² range: 0.25–0.46) as the aging associated gene signatures (Fig.1d). These genes exhibited positive correlations in their expression levels, with pairwise Spearman correlation coefficients ranging from 0.54–0.94. Furthermore, their expression levels consistently decreased across age decades (Fig. 1e). Using Over-representation analysis (ORA) based on Molecular Signatures Database (MSigDB)^13^, these 32 genes were significantly enriched in pathways such as IL-2/STAT5 signaling, TNF-α/NF-κB signaling, mTOR activation, complement activation, and cell-cycle checkpoint, reflecting processes such as inflammaging, immunosenescence, and metabolic rewiring (Fig. 1f). To evaluate the predictive power of these aging associated genes, we trained a regression model using their expression levels across the 4444 WBT samples and tested its performance in an independent validation cohort of 100 healthy individuals^14^. The model achieved a coefficient of determination (R²) of 0.31 and a mean absolute error (MAE) of 8.17 years (Supplementary Fig. 1b). To further benchmark its performance, we applied the same machine-learning pipeline and dataset to the 33 age-associated genes from the Korean Clock Project^15^, which resulted in R² = –0.14 and MAE = 13.77 (Supplementary Fig. 1c). This comparison highlights the strong predictive capacity of our WBT-derived aging gene signature.

### WBT captures health condition-specific transcriptomic changes

**Fig. 2:**
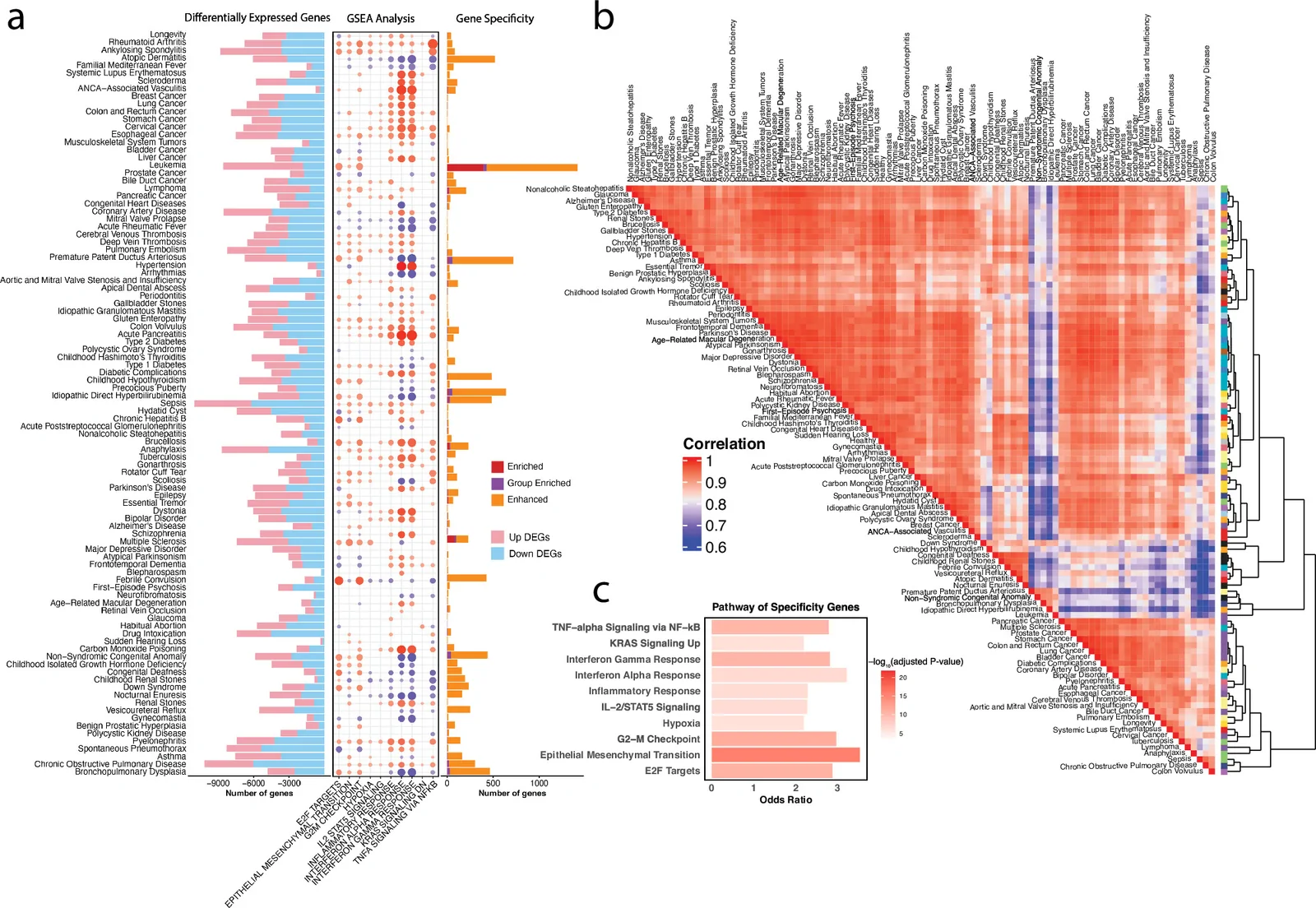
Gene and pathway features across health conditions. **a** Left: Number of significantly differentially expressed genes (DEGs; adjusted *P* < 0.05) for each health condition compared with healthy controls. Middle: Gene Set Enrichment Analysis (GSEA) of MSigDB pathways based on DEGs, illustrating whole-blood transcriptomics signatures across conditions. Right: Number of genes classified into “Disease Enriched,” “Group Enriched,” and “Disease Enhanced” categories. **b** Expression heatmap of the top 1000 most variable genes across all health conditions. Diseases were hierarchically clustered using a distance matrix defined as (1–Spearman correlation). **c** Top ten significantly enriched MSigDB Hallmark pathways for genes classified as “Disease Enriched”, “Group Enriched”, and “Disease Enhanced” across all health conditions. GSEA was performed using ranked gene lists. *P* values were adjusted for multiple comparisons using the Benjamini–Hochberg method. Source data are provided in the Source data file.

Next, we identified gene expression and pathway signatures across different health conditions. As shown in Fig. 2a, the number of significantly Differentially expressed genes (DEGs, adjusted *p* value < 0.05) compared to healthy controls varied across conditions. Notably, Sepsis, Chronic Obstructive Pulmonary Disease (COPD), Ankylosing Spondylitis, and Anaphylaxis exhibited the largest numbers of DEGs, whereas Blepharospasm showed the fewest (DEG results for all diseases are provided in Supplementary Data 1). To understand pathway-level differences across health conditions, we performed Gene Set Enrichment Analysis (GSEA) using MSigDB. Distinct pathway enrichment patterns were observed across the different health conditions. In rheumatoid arthritis and ankylosing spondylitis, TNF-α signaling via the NF-κB pathway was significantly upregulated. Besides, several cancer types exhibited elevated interferon gamma and interferon alpha responses. Together, these findings demonstrated that health condition-specific molecular signatures could be effectively captured at the WBT level.

We then classified disease-specific genes following the criteria defined by the Human Protein Atlas (HPA) (Supplementary Data 2). Leukemia displayed the highest number of disease-enriched and disease-enhanced genes, indicating a particularly distinct transcriptomic profile compared with other conditions. In total, 4,266 genes were classified as enriched, enhanced, or group-enriched across all health conditions (Supplementary Data 2). ORA revealed that these genes were predominantly associated with immune regulation and inflammatory response pathways, highlighting the central role of immune and inflammatory differences across health conditions (Fig. 2c).

We calculated pairwise Spearman correlations among all health conditions based on the mean expression levels of the 1000 most variable genes. Health conditions were then hierarchically clustered using a distance matrix defined as 1 minus the Spearman correlation (Fig. 2b). This approach grouped health conditions into different clusters. We observed that health conditions with shared biological mechanisms tended to cluster together. For example, cancers formed a coherent cluster, as did neurological disorders. In contrast, conditions such as sepsis and COPD showed lower similarity to other diseases, reflecting their distinct and highly systemic inflammatory profiles.

### Cell-type composition and metabolic changes across disease

**Fig. 3:**
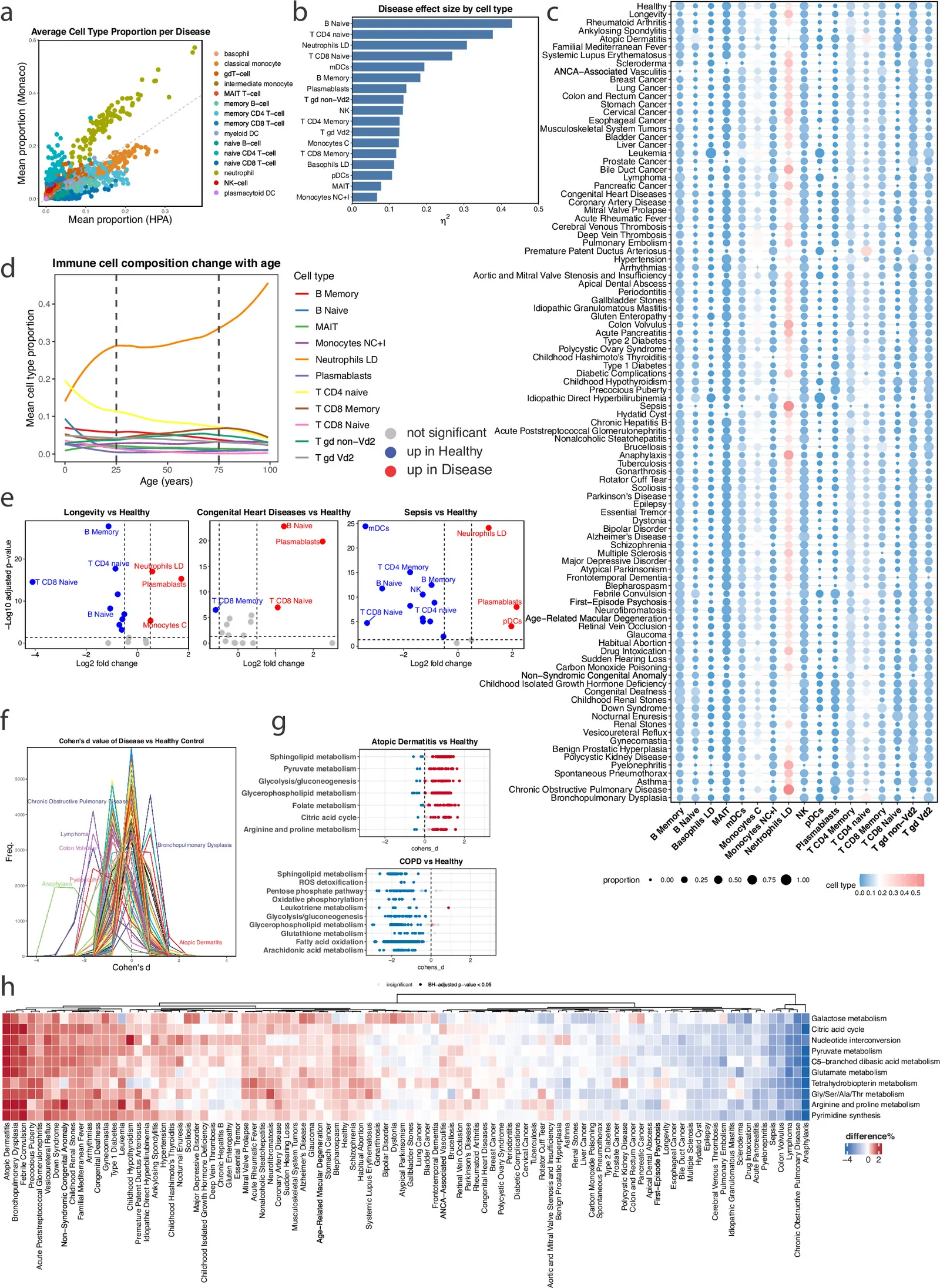
Immune cell composition and metabolic diversity across health conditions. **a** Comparison of immune cell proportions estimated using the HPA and Monaco reference datasets for deconvolution. Each point represents the mean proportion of a specific immune cell type within a health condition. **b** Effect sizes (η²) of disease status on the proportion of each immune cell type, estimated by one-way ANOVA. **c** Bubble plot showing mean immune cell proportions inferred by CIBERSORTx deconvolution using the Monaco reference. Color indicates the percentage of each immune cell type within a disease, while bubble size reflects normalized cell fractions across diseases, enabling cross-condition comparison. **d** Eleven immune cell types were significantly associated with age. Their age-dependent trajectories are shown, revealing two major inflection points at ~25 years and 75 years, where immune cell proportions undergo the most pronounced changes. **e** Comparative analysis of immune cell fractions in sepsis, longevity, and congenital heart disease compared with healthy controls. Each immune cell type was compared with healthy controls using a two-sided Wilcoxon rank-sum test. *P* values were adjusted for multiple comparisons using the Bonferroni method. Red indicates significantly increased immune cell fractions, whereas blue indicates significantly decreased immune cell fractions. **f** Distribution of Cohen’s d statistic of metabolic activity across different diseases. The curves describe the number of metabolic reactions that are significantly altered (COMPASS analysis, FDR < 0.05) in each disease relative to healthy controls. Downregulated reactions dominate Anaphylaxis and COPD, whereas Atopic Dermatitis exhibits the highest number of upregulated reactions. **g** Metabolic activity changes in representative metabolic subsystems for atopic dermatitis versus healthy controls and COPD versus healthy controls, assessed using the Wilcoxon test. COPD is characterized by widespread downregulation of energy-related reactions, whereas atopic dermatitis shows extensive upregulation, particularly in amino acid metabolism. **h** Disease-specific metabolic activity profiles across all health conditions. Heatmap displays z-score normalized metabolic pathway activity scores across health conditions, generated by COMPASS analysis of a global genome-scale metabolic model (Recon3D) integrated with transcriptomic data. Source data are provided in the Source data file.

We applied CIBERSORTx, a machine learning-based deconvolution algorithm, to estimate immune cell proportions in WBT using two reference blood immune cell RNA-seq datasets: the HPA immune cell dataset^16^ and the Monaco dataset^17^. At the disease level, mean proportions estimated by the two references showed a positive overall relationship (Fig. 3a), although the degree of concordance varied by cell type, with high-abundance cell types (e.g., neutrophils, classical monocytes) showing the strongest agreement. The cell-type proportions for each disease are shown in Fig. 3c (Monaco) and Supplementary Fig. 2a (HPA). T CD4 naive, B naive, and neutrophil cells showed the strongest disease-associated effects on cell type proportion (Fig. 3b). The proportions of these cell types exhibited significant alterations in many conditions (Supplementary Fig. 2b–d). We identified significant age-associated shifts in immune cell composition (Fig.3d), with 11 cell types showing robust increases or decreases in proportion after adjusting for disease status. Neutrophils showed a consistently increasing trend with age, whereas T CD4 naïve cells declined steadily. Interestingly, the most pronounced shifts in cell proportions occurred around ages 25 and 75, while the interval between these ages was relatively stable. In longevity participants (age >80), we observed significantly higher proportions of neutrophils and plasmablasts, along with reduced proportions of naive T and B cells, compared with healthy controls whose mean age was 39.3 years (Fig. 3e). In contrast, congenital heart disease was characterized by higher proportions of naïve B cells and naïve CD8 T cells, along with a lower proportion of CD8 memory T cells. This pattern was consistent with the younger age of this disease group (mean age = 10.1). In sepsis patients, increased neutrophil proportions accompanied by marked lymphocyte reduction were consistent with the neutrophilia and lymphopenia characteristic of the acute sepsis response.

Metabolic enzymes are encoded by genes, enabling the study of metabolic features using transcriptomic data^18,19^. We used COMPASS^20^ to infer metabolic activity across different diseases based on the generic human genome-scale metabolic model, RECON3, identifying the activity of 6135 reactions across 95 metabolic subsystems. Among all metabolic subsystems, those related to energy production, such as galactose metabolism, the citric acid cycle, nucleotide interconversion, and pyruvate metabolism, showed the greatest variation across health conditions. We selected the ten subsystems with the highest variance and visualized their metabolic activity patterns across different health conditions. As shown in Fig. 3h, anaphylaxis, COPD, pulmonary embolism, cerebral venous thrombosis, and sepsis, conditions associated with circulatory insufficiency, impaired oxygen delivery, or immune suppression, exhibited globally downregulated metabolic activity. In contrast, atopic dermatitis and several pediatric diseases showed markedly elevated metabolic activity, reflecting their high energy-demanding and immunologically activated states. Healthy individuals and psychiatric disorders were positioned near the center of the metabolic spectrum, indicating relatively stable systemic metabolic activity compared with other conditions. We then compared the metabolic activity of each health condition with that of the healthy group. As shown in Fig. 3f, anaphylaxis and COPD exhibited the highest numbers of downregulated reactions, whereas atopic dermatitis displayed the greatest number of upregulated reactions. We then selected atopic dermatitis and COPD as two representative conditions for further analysis. In atopic dermatitis, reactions associated with arginine and proline metabolism, glycosphingolipid metabolism, and several related pathways were significantly upregulated (Fig.3g), representing the characteristic metabolic alterations observed in these patients^21^. In contrast, COPD showed a markedly different pattern: reactions involved in oxidative phosphorylation, glycolysis, glutathione metabolism, and fatty acid oxidation were all significantly downregulated. These findings were consistent with previous studies reporting metabolic dysregulation in energy^22^, lipid, and amino acid metabolism^23^ in patients with COPD.

### WGCNA identifies cell type-specific gene modules

Since genes often function within coordinated networks, after identifying signatures at both the gene, pathway, cell-type, and metabolic levels, we next examined health condition-specific transcriptomic patterns through the analysis of gene co-expression clusters using Weighted Gene Co-expression Network Analysis (WGCNA)^24^. This unsupervised method begins by calculating pairwise correlations between all gene pairs and converting the resulting matrix into a scale-free network. Genes with highly correlated expression patterns are then grouped into distinct co-expression clusters using hierarchical clustering.

**Fig. 4:**
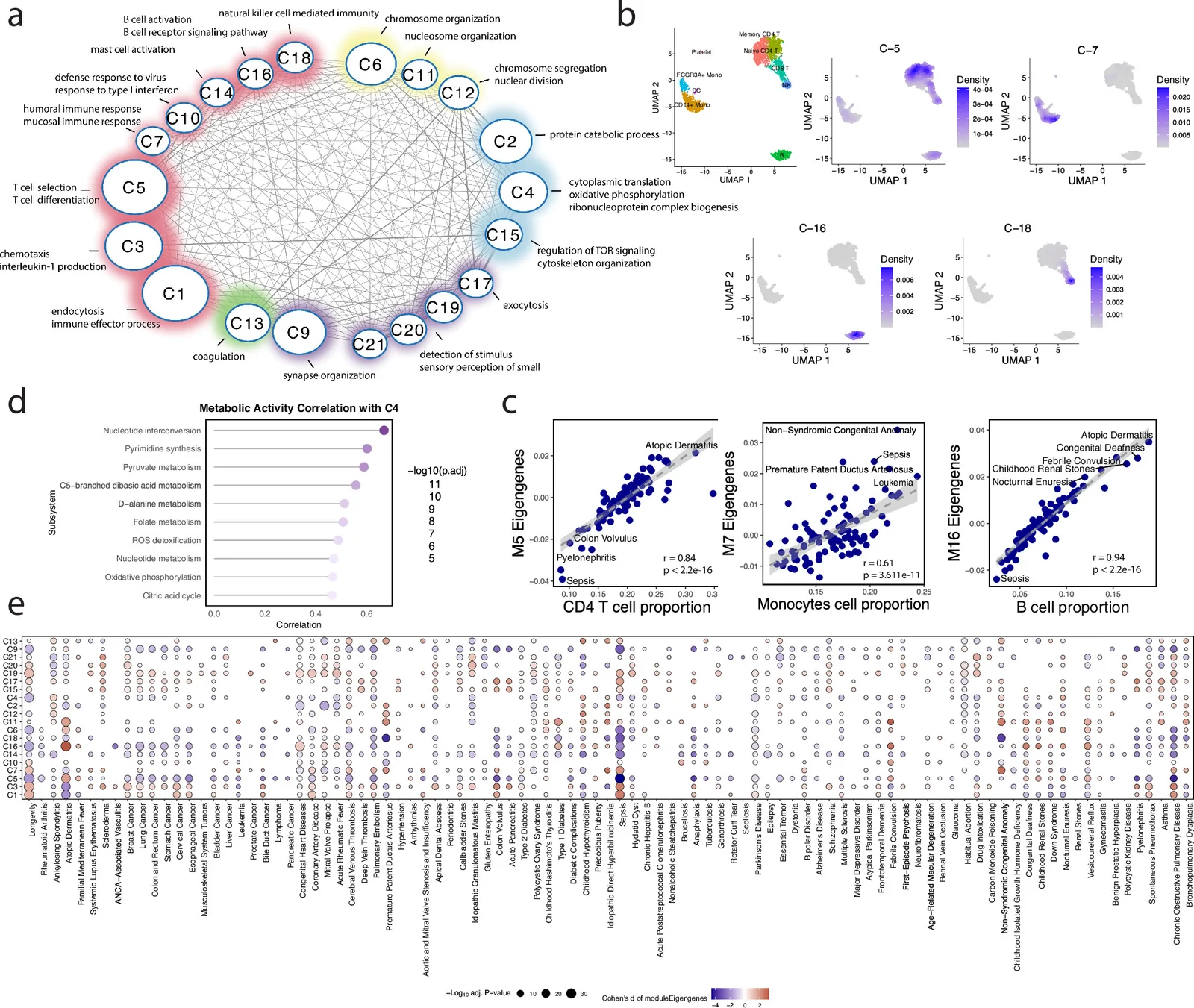
Gene co-expression modules reflecting cell-type–specific and functional features. **a** Weighted Gene Co-Expression Network Analysis (WGCNA) of whole blood transcriptomic data from 4444 participants identified 21 co-expression gene clusters with significant functional enrichment. Node size represents the number of genes per module, and edge thickness indicates the strength of inter-module correlations. The clusters exhibited distinct biological functions: red clusters were associated with cytokine signaling and inflammatory responses; yellow clusters were enriched for cell-cycle and proliferative processes; blue clusters reflected metabolic and mitochondrial pathways; purple clusters were linked to signal transduction; and green clusters were related to tissue repair. Functional annotations for each module are labeled in the figure. **b** UCell enrichment analysis based PBMC single-cell data confirmed the cell-type–specific enrichment patterns of distinct cluster, consistent with GO-defined cell-type characteristics. Cluster 5 showed strong enrichment in T cells, Cluster 7 in monocytes, Cluster 16 in B cells, and Cluster 18 in NK cells. **c** Immune-cell–specific gene modules showed strong correlations with the corresponding cell proportions inferred by deconvolution (two-sided Pearson’s correlation test, r = 0.84, 0.61, and 0.94 for CD4 + T cells, monocytes, and B cells, respectively). The dashed line represents the linear regression fit, and the shaded area indicates the 95% confidence interval. **d** C4, representing metabolic and mitochondrial processes, was strongly associated with energy-production metabolic activity predicted by the genome-scale metabolic model. Two-sided Pearson’s correlation test (*n* = 95 subsystems tested, BH-adjusted); top 10 subsystems shown, colored by −log10(adjusted *p* value). **e** Cluster eigengene expression across health conditions (two-sided Wilcoxon rank-sum test, BH-adjusted *p* values). Bubble plot shows Cohen’s d effect sizes for each disease vs. healthy controls; dot size reflects −log10 adjusted *p* value. Source data are provided in the Source data file.

Using this framework, we identified 21 gene co-expression clusters based on the WBT profiles of 4444 participants (genes in each cluster are listed in Supplementary Data 3). To characterize the biological functions of each cluster, we performed ORA using the Gene Ontology (GO) database, which enabled the identification of significantly enriched biological pathways. In total, 20 clusters showed significant functional enrichment. The top five GO-enriched pathways for each cluster were provided in Supplementary Fig. 3. We found that the functional profiles of the clusters could be classified into five major categories (Fig. 4a): cytokine signaling and immune response (red), cell cycle and proliferation (yellow), metabolism and mitochondrial processes (blue), signal transduction (purple), and tissue repair (green). Interestingly, we also observed cell-type-specific immune signatures within several clusters. For example, Cluster 5 was associated with T-cell activation, Cluster 16 was linked to B-cell activation, and Cluster 18 was enriched for natural killer (NK) cell–mediated immunity. To further assess the cell-type specificity of the gene clusters, we analyzed a healthy donor peripheral blood mononuclear cell (PBMC) single-cell RNA-seq dataset. This dataset contained eight major immune cell types, including Naive CD4 T cells, CD14⁺ monocytes, Memory CD4 T cells, B cells, CD8 T cells, FCGR3A⁺ monocytes, NK cells, dendritic cells (DCs), and platelets. We then applied UCell to quantify the enrichment of these cell-type–specific clusters within the single-cell PBMC dataset. As shown in Fig. 4b, cluster 5 showed strong enrichment in T cells, cluster 7 was enriched in monocytes, cluster 16 displayed specific enrichment in B cells, and cluster 18 was enriched in NK cells. We further correlated the module eigengenes (MEs) values of these cell-type–associated clusters with the corresponding immune-cell proportions derived from WBT deconvolution. These relationships again showed high concordance, with Pearson correlation coefficients of r = 0.84, 0.61, and 0.94 for CD4 T cells, monocytes, and B cells, respectively (Fig. 4c). In addition, cluster 4, which represents metabolism and mitochondrial processes, was closely associated with energy-production metabolic activity identified from the metabolic model (Fig. 4d). Together, these findings demonstrate that the gene clusters we identified capture distinct biological processes, and that their functional characteristics can be validated consistently across multiple analytical perspectives.

After confirming the biological functions of each cluster, we further explored their expression relevance across different health conditions by analyzing the MEs. For each gene cluster, ME values across disease groups were compared to those in healthy controls. Effect sizes were calculated using Cohen’s d. Statistical significance was evaluated using the Wilcoxon rank-sum test. As shown in Fig. 4e, the expression patterns of gene clusters varied across health conditions, and the gene co-expression profiles effectively reflected disease-specific characteristics. For example, in the longevity group, T-cell–related (C5) and B-cell–related modules (C16) showed marked declines, whereas innate inflammatory modules (C1, C3, C7, and C10) tended to increase, reflecting the combined effects of immunosenescence and inflammaging^25,26^. For COPD and sepsis, the T-cell (C5), mast-cell (C14), B-cell (C16), and NK-cell (C18) clusters were all decreased. These findings highlight cell-type–specific immune alterations across different disease conditions.

### RNA–protein modules reveal complementary omics regulation

**Fig. 5:**
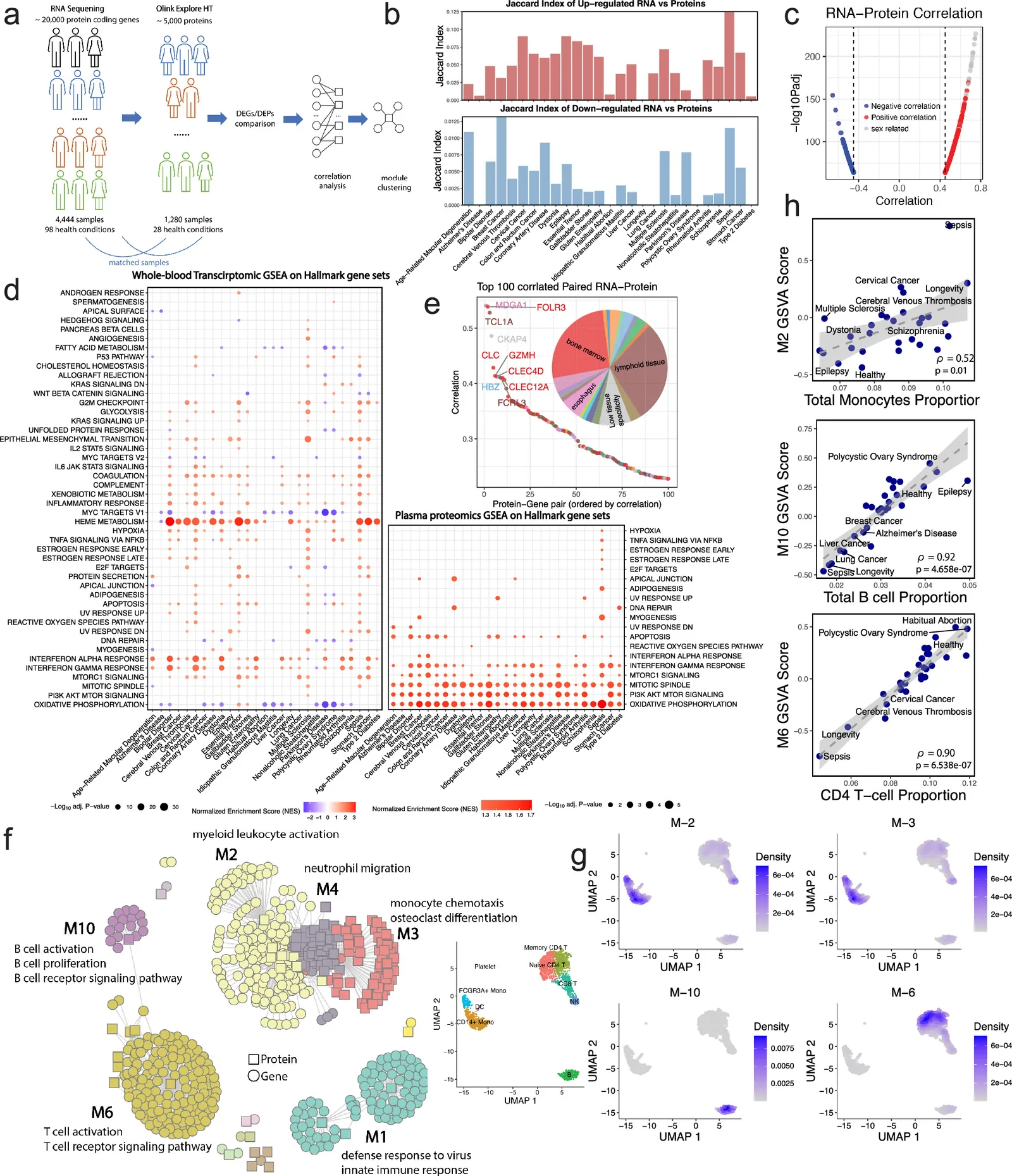
Immune cell–specific gene–protein correlation. **a** In addition to 4444 whole-blood transcriptomes, we generated matched proteomic profiles for 1280 samples across 28 health conditions using Olink Explore HT. Differentially expressed genes (DEGs) and proteins (DEPs) were identified relative to healthy controls, followed by integrated RNA–protein correlation and network analyses. **b** The Jaccard index showed limited overlap between DEGs and DEPs across diseases, ranging from 0 to 0.13. **c** Correlations between ~20,000 protein-coding genes and ~5000 proteins identified RNA–protein pairs with Pearson’s r > 0.45 (two-sided test, BH-adjusted). The strongest associations were sex-related and were excluded from subsequent network analyses. **d** Gene set enrichment analysis (GSEA) revealed that transcriptomics identified more significantly altered pathways than proteomics. Most pathways showed consistent enrichment across both modalities, whereas some, such as oxidative phosphorylation, displayed opposite directions, highlighting post-translational regulation and the complementary value of transcriptomic and proteomic profiling. **e** The top 100 RNAs showing the strongest correlations with their corresponding proteins were predominantly associated with bone marrow and lymphoid tissues, indicating that whole-blood transcriptomic profiles are closely linked to immune cell–derived proteins. **f** Pairwise RNA–protein correlations were integrated into a multi-edge network, and Louvain clustering identified several RNA–protein modules. GO enrichment analysis was performed to annotate the biological pathways associated with the RNAs in different modules. **g** UCell enrichment scores of the RNA-protein modules across eight immune cell types in a single-cell PBMC dataset. Module 2 and Module 3 showed strong enrichment in monocytes, Module 10 in B cells, and Module 6 in CD4 + T cells. **h** Correlations between GSVA scores of RNA–protein modules and cell type proportions derived from WBT deconvolution were high, further confirming cell-type–specific features across different diseases (two-sided Spearman’s rank correlation test). The dashed line represents the linear regression fit, and the shaded area indicates the 95% confidence interval. Source data are provided in the Source data file.

To explore the relationship between WBT and plasma proteomics, we analyzed 1280 samples with Olink Explore HT data across 28 health conditions (Fig.5a). We first compared DEGs and differentially expressed proteins (DEPs) for each disease relative to healthy controls to assess whether RNA- and protein-level perturbations exhibited consistent patterns. As shown in Fig. 5b, the overlap between DEGs and DEPs was limited across diseases, with Jaccard indices for both up- and down-regulated sets below 0.13, indicating that RNA and protein levels capture distinct perturbation patterns. We further performed GSEA based on DEGs and DEPs (Fig. 5d). At the RNA level, more significantly altered pathways were identified, suggesting that WBT captures broader perturbations than plasma proteomics. For overlapping pathways, some exhibited consistent regulation at both levels; for example, interferon gamma response was upregulated at both the RNA and protein levels. In contrast, other pathways, such as oxidative phosphorylation, showed opposite trends, implying the influence of post-transcriptional regulation. Together, these findings highlight that RNA and protein expression reflect different aspects of disease biology and provide complementary information.

Next, we conducted RNA-protein correlation analysis. We first examined correlations between RNAs and their corresponding proteins (Fig. 5e). Using the HPA database to annotate tissue specificity, we found that highly concordant RNA–protein pairs were predominantly specific to bone marrow and lymphoid tissues. This suggests that immune cell–derived RNA and their corresponding secreted or surface proteins are more tightly coupled in blood than those derived from other tissue sources.

Across all gene–protein pairs (19,314 RNAs × 5348 proteins), we identified 3523 pairs with strong positive Pearson correlations (r > 0.45; Fig. 5c). Notably, Y chromosome–linked genes and proteins enriched in male reproductive organs showed the strongest correlations (e.g., UTY–EDDM38, r = 0.74), underscoring the robustness and reliability of the results. To focus on biologically relevant associations, these sex-related pairs were removed prior to downstream analysis. The remaining 3480 pairs were integrated into a multi-edge RNA–protein network, which was subsequently partitioned into distinct regulatory modules using the Louvain clustering algorithm (Fig. 5f). The RNA and protein members of each module are listed in Supplementary Data 4. The function of each module was annotated using the GO database, which revealed distinct immune cell–related features. For example, module 6 was associated with T cell activation, module 10 with B cell activation, module 3 with mononuclear cell differentiation, and module 1 with innate immune responses. We further mapped the RNAs from these modules to the single-cell PBMC dataset (Fig. 5g). Consistently, RNAs in M6 were enriched in CD4 T cells, those in M10 in B cells, and those in M2 and M3 in CD14⁺ monocytes. The enrichment of module-associated RNAs across health conditions was assessed using Gene Set Variation Analysis (GSVA). When we correlated module enrichment features with the corresponding immune cell proportions across all health conditions, we observed strong associations (Fig. 5h), further demonstrating the high immune-cell specificity of these RNA–protein modules.

**Fig. 6:**
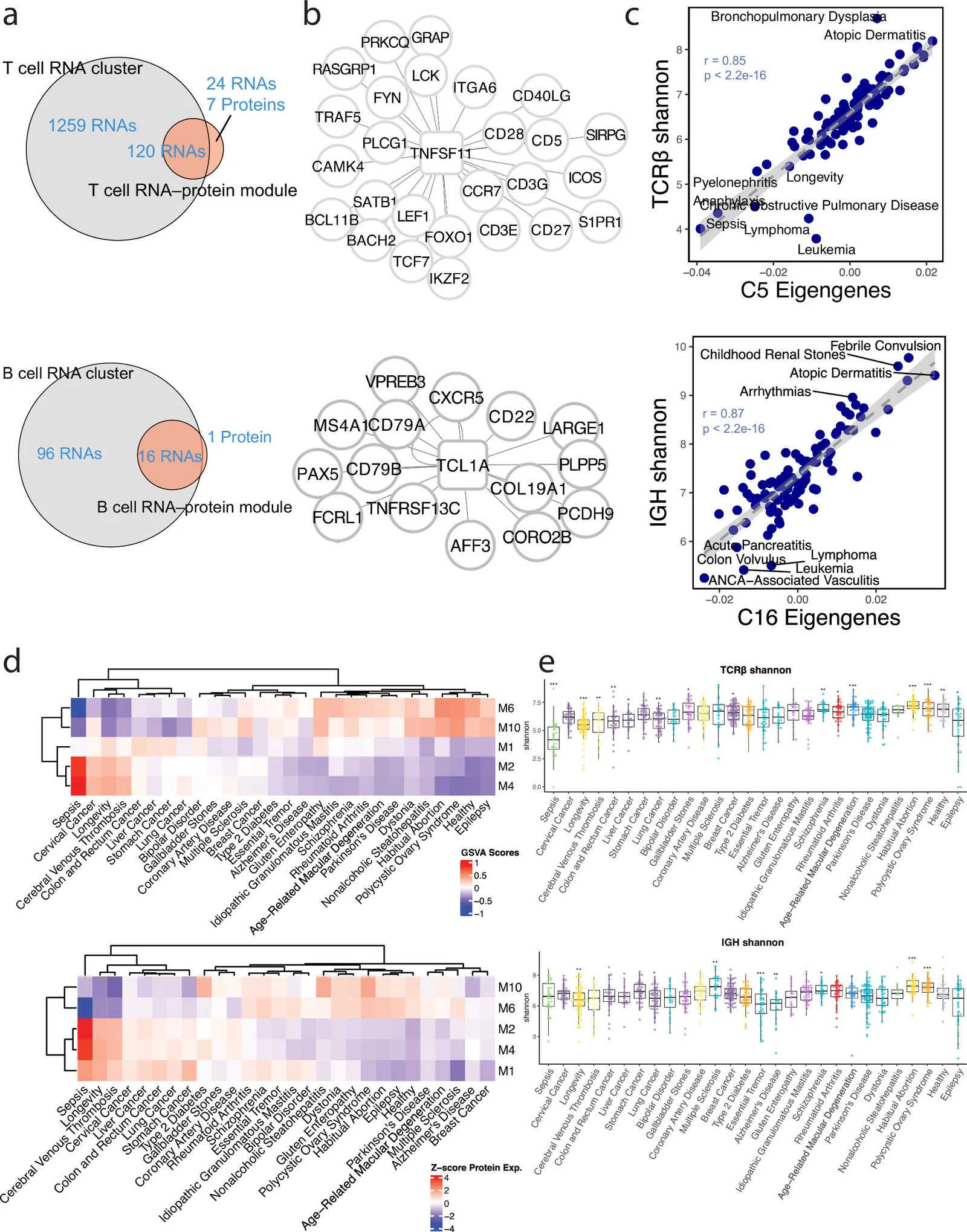
T-Cell and B-cell RNA–protein module features. **a** Venn diagrams showing the substantial overlap between T-cell and B-cell RNA clusters and their corresponding RNA–protein modules, highlighting the central role of the identified features. **b** Network visualization of representative RNAs and proteins from the T-cell and B-cell modules, respectively. **c** The eigengenes of the T-cell and B-cell clusters showed strong correlations with the Shannon diversity indices of TCRβ and IGH, respectively (two-sided Pearson’s correlation test), demonstrating the immune-specific characteristics of these clusters. The dashed line represents the linear regression fit, and the shaded area indicates the 95% confidence interval. **d** Heatmaps of GSVA scores for RNAs within each module at the disease level, alongside the mean protein expression levels of their corresponding modules, highlighting disease-specific molecular **e** Shannon diversity index of TCRβ and IGH repertoires across multiple disease states. Each dot represents one biologically independent participant. The center line represents the median; the box limits indicate the first and third quartiles (25th and 75th percentiles); whiskers extend to the most extreme data points within 1.5 × the interquartile range. Each disease group was compared with all remaining disease groups using a two-sided Wilcoxon rank-sum test; *p* values were adjusted for multiple comparisons using the Benjamini-Hochberg (BH) method. Significance levels: *** *p* < 0.001, ** 0.001 <*p* < 0.01, * 0.01 <*p* < 0.05. Sample sizes for each disease group, exact adjusted *p* values, and all other source data are provided in the Source Data file.

Since both immune cell–related RNA clusters and RNA–protein modules were identified, we examined the overlap between the two layers. As shown in Fig. 6a, most RNAs within these RNA–protein modules also appeared in the corresponding RNA clusters, highlighting their central role in immune cell regulation. We further visualized the key RNAs and proteins within the T cell and B cell RNA–protein modules to illustrate their internal structure (Fig. 6b). Besides, to assess immune characteristics more comprehensively, we evaluated the T cell receptor (TCR) and B cell receptor (BCR) repertoires. We calculated the Shannon index for TCRβ and IGH as a measure of T cell and B cell diversity. As shown in Fig. 6c, the expression levels of the T cell and B cell clusters were highly correlated with the Shannon index of the corresponding immune cell type. We also found RNA and protein expression patterns within each module across different health conditions (Fig. 6d). Notably, patients with sepsis exhibited a distinct module profile, characterized by reduced expression of the T cell module (M6), whereas patients with habitual abortion showed increased expression of this module. These observations were further supported by TCR analysis (Fig. 6e), which revealed markedly decreased TCR diversity in sepsis and increased diversity in habitual abortion.

### Multi-disease classification by transcriptomic and proteomic signatures

**Fig. 7:**
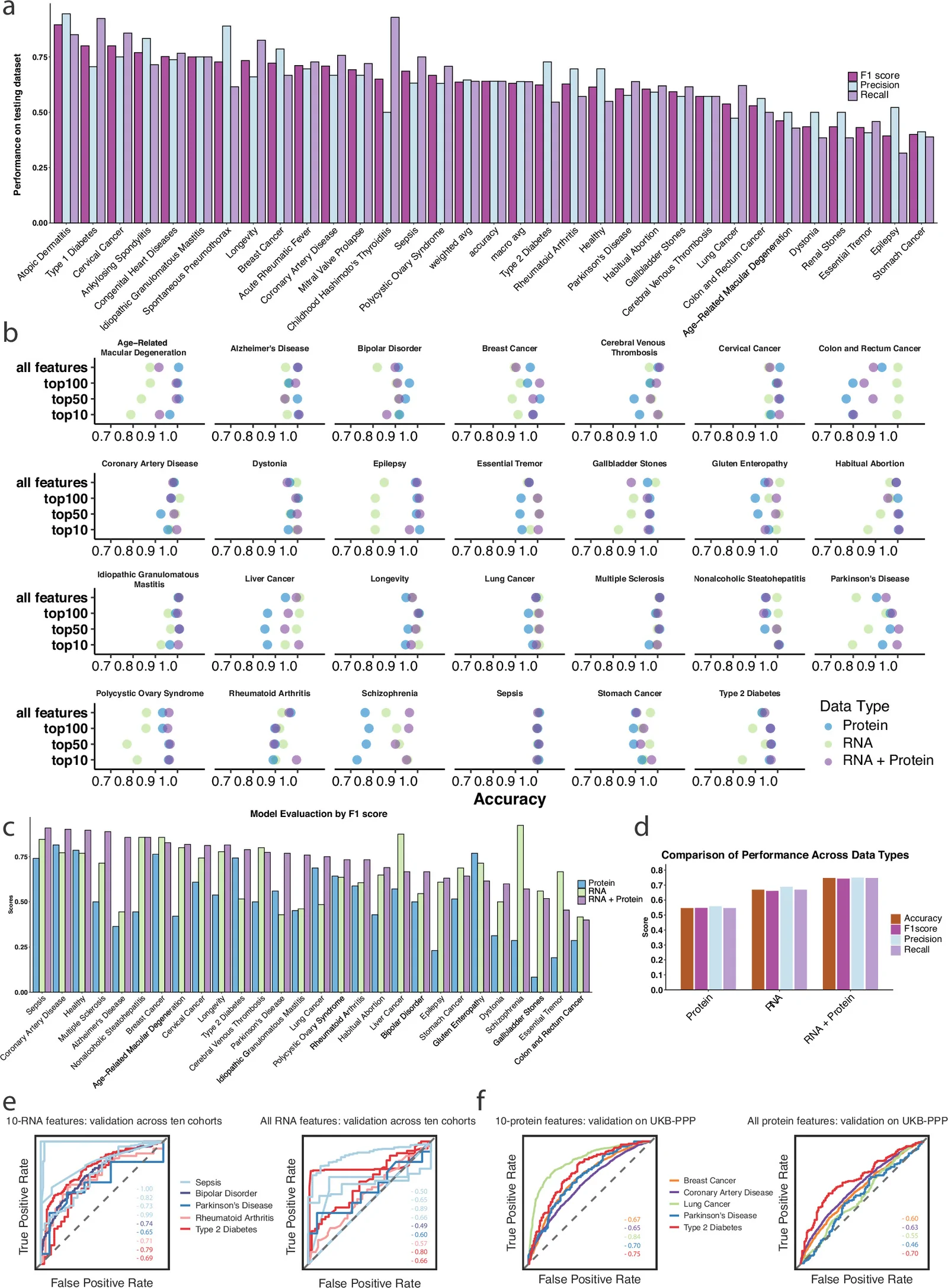
Machine learning–based disease prediction using disease-specific signatures. **a** F1 score, precision, and recall were used to evaluate class-specific performance of the multi-class model trained on WBT data across 29 health conditions. **b** Binary-classification performance of RNA, protein, and combined RNA–protein features in distinguishing each disease from healthy controls. For each disease, four feature sets were tested (top 10, top 50, top 100 SHAP-ranked features, and all features). The results show that the top 10 features retained strong predictive power, performing comparably to much larger feature sets. **c** Comparison of RNA, protein, and combined RNA–protein datasets in a multi-class model distinguishing 28 health conditions using the same training pipeline. Integrating RNA and protein markedly improved predictive performance. **d** Mean predictive performance of the three data types (RNA, protein, and RNA–protein) in the multi-class model. **e** Machine learning classifiers trained on the top 10 SHAP-ranked disease-specific RNA features and tested on ten independent WBT cohorts comprising 1621 samples achieved a mean AUC of 0.79. As a benchmark, models trained using all RNA features with the same pipeline showed reduced performance (AUC = 0.65) (**f**). Protein-based classifiers evaluated on the UKB-PPP cohort (9998 samples) achieved a mean AUC of 0.73. Benchmark models trained using all protein features showed lower performance (AUC = 0.59), further demonstrating the predictive strength of the selected signatures. Source data are provided in the Source data file.

After exploring cell–type–specific, immune, and metabolic features at the gene, pathway, gene cluster, as well as RNA-protein module levels, we found that WBT could effectively capture disease features. We then selected diseases with more than 40 samples for machine learning based classification. The entire cohort was randomly divided into a training set (70%, *n* = 2079) and a testing set (30%, *n* = 891) across 29 health conditions. Feature importance for each RNA was assessed using SHAP in the training dataset. We first distinguished each disease from healthy controls using binary classifiers and compared model performance on the testing dataset when using the top 10, top 50, top 100, or all RNAs as features. For each classification task, the best-performing model was selected based on five-fold cross-validation in the training data and subsequently evaluated on the testing data. As shown in Supplementary Fig. 4a, models built on the top 10 SHAP-ranked RNAs achieved performance comparable to those using larger feature sets, with testing AUC values ranging from 0.89–1.0. Using the disease-specific sets of 10 RNAs for each condition in a multi-class model, we achieved an overall F1 score and accuracy of 0.64 across 29 health conditions (Fig. 7a). To determine whether disease-associated transcriptomic differences reflect intrinsic gene expression changes or cell composition shifts, we compared three models with the same pipeline: RNA only (top 10 SHAP genes), cell type proportions only, and both combined. Incorporating cell proportions did not improve predictive performance over the RNA-only model (Supplementary Fig. 5).

**Fig. 8:**
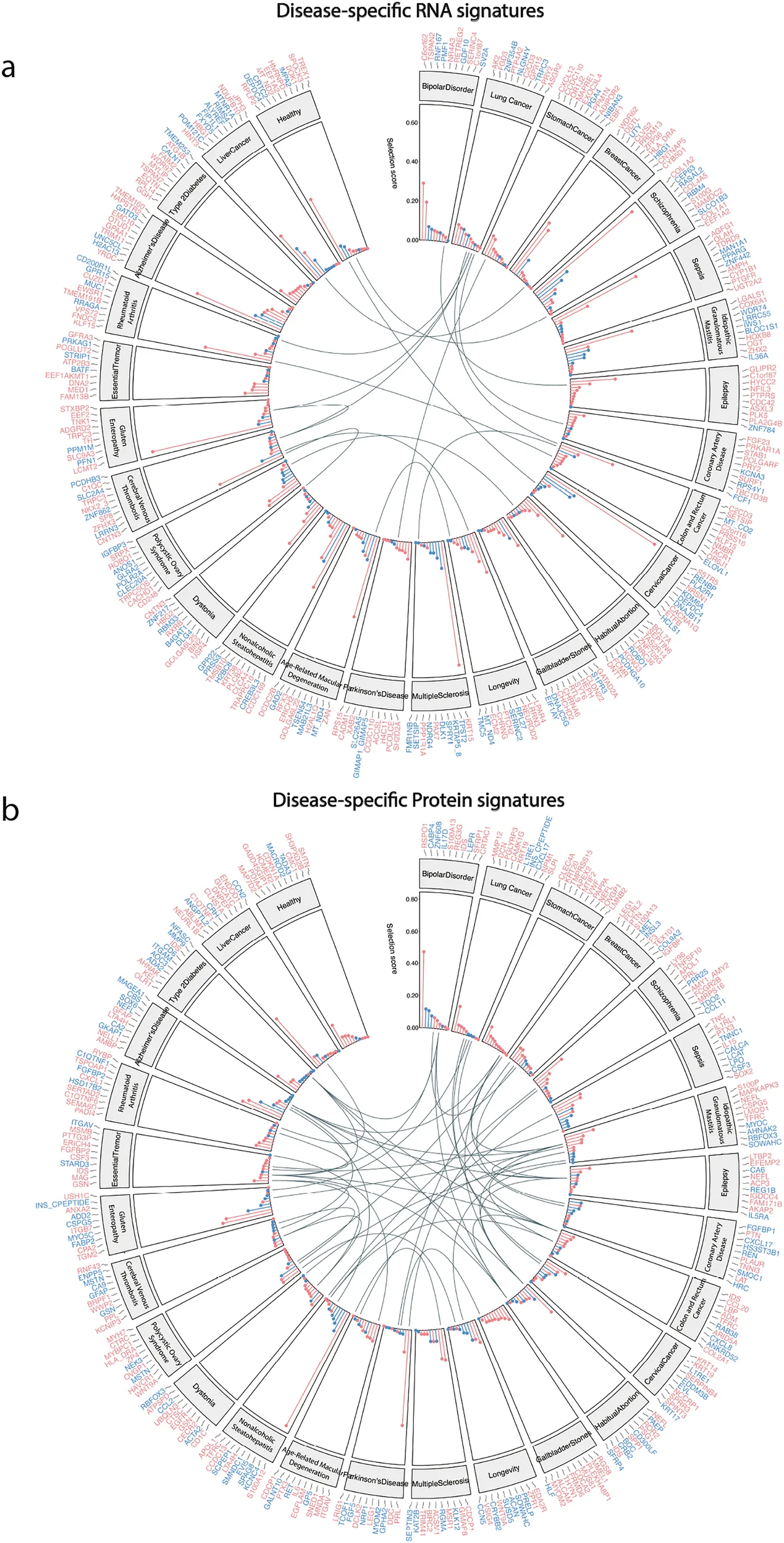
Circular chord diagram of the top 10 disease features across health conditions. Circular chord diagrams showing the top 10 disease-specific signature RNAs (**a**) and proteins (**b**) for each health condition. Bar length represents the SHAP importance score. Features that push the model’s prediction toward the positive class are shown in red, whereas those pushing it toward the negative class are shown in blue. Shared genes or proteins across conditions are connected by curves in the center of the diagram. Source data are provided in the Source data file.

As our results indicated the complementary nature of blood RNA and protein features, we next evaluated model performance using single-omics data versus their combination. To ensure comparability, we focused on the 1280 matched samples across 28 health conditions and split them into a training set (70%, *n* = 896) and a testing set (30%, *n* = 384). We first assessed binary classification performance by distinguishing each disease from healthy controls. As shown in Fig. 7b, integrating RNA and protein generally yielded the highest predictive performance across diseases. However, some conditions were better predicted by protein alone (e.g., bipolar disorder), whereas others were better captured by RNA (e.g., colon and rectal cancer). For sepsis, which exhibits a strong blood-based signal, all data types—RNA, protein, or combined—achieved similarly high performance. Next, we compared the performance of multi-class models built on different data types. For feature selection, the top 10 signature RNAs or proteins for each disease were identified using SHAP scores derived from binary classifiers that distinguished each health condition from the remaining samples in the training dataset. This procedure identified 264 unique RNA signatures and 244 unique protein signatures in total. No overlapping features were observed between the two data types, further highlighting that RNA and protein capture distinct aspects of disease biology. The resulting RNA and protein signatures for each disease are shown in Fig. 8. Many of these signatures exhibit immune cell or tissue specificity, as detailed in Supplementary Data 5.

Model performance based on these selected features was first evaluated on the training dataset using nine different machine-learning algorithms under stratified 10-fold cross-validation, with weighted F1 score as the optimization metric. The best-performing model for each data type was then subjected to hyperparameter tuning before being applied to the testing dataset. Following this pipeline, we trained multi-class models for RNA alone, protein alone, and RNA–protein combined data. For the protein-only model, logistic regression was the best-performing classifier during cross-validation on the training dataset and achieved an F1 score of 0.55 and an accuracy of 0.55 on the testing dataset. For the RNA-based model, the random forest classifier performed best during training and reached an F1 score of 0.66 and an accuracy of 0.67 on testing. When RNA and protein features were combined, logistic regression again produced the highest performance during cross-validation, yielding an F1 score of 0.74 and an accuracy of 0.75 on the testing dataset (Fig. 7d). Class-level performance for each multi-class model is presented in Fig. 7c and Supplementary Fig. 4b, c. To ensure that performance differences were not driven by unequal feature dimensionality across data types, we also evaluated models using the top 20 RNA signatures, the top 20 protein signatures, and a combined feature set consisting of the top 10 RNA plus top 10 protein signatures. The combined feature set again produced the strongest predictive accuracy, with F1 scores of 0.516 (protein-only), 0.624 (RNA-only), and 0.743 (RNA+protein) (Supplementary Fig. 6).

After confirming that the signature RNAs and proteins effectively distinguished diseases in the internal dataset, we further evaluated their generalizability in independent validation cohorts. For transcriptomic validation, we collected ten external cohorts comprising a total of 1621 samples across five diseases: sepsis, bipolar disorder, Parkinson’s disease, rheumatoid arthritis, and type 2 diabetes. Using the disease-specific sets of 10 signature RNAs identified in our training data, we trained binary classifiers on our dataset and assessed their performance in each independent cohort. Following model selection and hyperparameter tuning on the training dataset, the resulting models achieved a mean AUC of 0.79 and a mean balanced accuracy of 0.73 across the external cohorts. To benchmark the predictive power of the selected signature, we applied the same machine learning pipeline—with identical model selection and hyperparameter tuning procedures—using PCA-derived features from all RNAs. These benchmark models performance resulted in a mean AUC of 0.65 and a mean balanced accuracy of 0.56 (Fig. 7e).

For proteomic validation, we included diseases with more than 50 samples in our dataset and identified the corresponding conditions in the UKB-PPP cohort. This yielded five diseases—breast cancer, coronary artery disease, lung cancer, Parkinson’s disease, and type 2 diabetes—with a total of 9998 samples. Using the disease-specific signature proteins derived from our dataset, the models achieved a mean AUC of 0.73 in the UKB cohort. In contrast, applying the same pipeline but using all proteins as features resulted in a mean AUC of 0.59 (Fig. 7f).

Detailed performance metrics, including the selected models, accuracy, balanced accuracy, and F1 scores for each RNA and protein classifier in the machine learning section, are provided in Supplementary Data 6. Together, these results demonstrate that the disease-specific RNA and protein signatures identified from our internal dataset remain robust and predictive in independent external cohorts.

## DISCUSSION

Here, we present the WBT Atlas, which includes a cohort of 4444 participants across 98 health conditions (https://www.phenomeatlas.org/wbt/). In this study, we began by examining the influence of demographic factors on gene expression profiling. Variance analysis revealed that health status was the most influential factor shaping WBT variation, providing a foundation for subsequent disease-specific investigations. Building on this, we characterized distinct gene expression and pathway signatures associated with individual health conditions. Recognizing that genes often function in coordinated networks, we further identified gene co-expression clusters shared across the population. Notably, several clusters exhibited apparent immune cell-type specificity, as validated by cell-type deconvolution and analyses of immune cell diversity. Furthermore, our cohorts included 1280 participants with matched proteomics data. Through RNA–protein correlation analysis, we identified immune cell–specific gene–protein regulatory relationships, highlighting the complementary features of WBT and plasma proteomics. We also built a multi-class disease classification model based on the disease-specific RNA and protein signatures, achieving robust predictive performance. We finally validated the generalizability of these signatures using ten independent WBT cohorts and the UKB-PPP dataset. Our analyses revealed that disease-specific signatures are evident not only at the gene and pathway levels but also in co-expression modules and immune cell proportions. These dual layers of information, reflecting intracellular regulatory activity and systemic immune landscape, converge to form health condition-specific transcriptomic profiles. Thus, the WBT Atlas serves as a proxy for systemic physiological states and complements plasma proteomics, making it a powerful, minimally invasive tool for detecting a wide range of health conditions.

WBT Atlas captures the total RNA content present in the whole blood, encompassing multiple RNA sources: (i) the immune cells, including leukocytes, such as neutrophils and lymphocytes; (ii) erythroid cells with hemoglobin transcripts (e.g., *HBA1, HBA2, HBB*); (iii) platelet-derived cells; and (iv) circulating cell-free RNA (cfRNA). Among these, leukocytes contribute the majority of RNA in WBT. A previous study demonstrated that the combined counts of neutrophils and lymphocytes alone could explain up to 75% of the variation in protein-coding gene expression in peripheral blood^27^. Hemoglobin transcripts can account for a substantial proportion of total RNA (52-76%), potentially masking low-abundance transcripts^28^. To mitigate this issue and improve data sensitivity and specificity, we employed a globin depletion step during library preparation, which effectively removed globin RNA. Besides, transcripts from platelet-derived RNA and cfRNA could be detected in WBT^29,30^. Although present at relatively low abundance, these RNA species carry biologically meaningful signals related to platelet activation, immune modulation, and tissue-derived processes. Overall, WBT provides a comprehensive snapshot of systemic gene expression, making it particularly well-suited for large-cohort studies and biomarker discovery. An important consideration in WBT analysis is whether observed transcriptomic differences reflect changes in cell type composition or intrinsic gene expression changes within cells. By comparing predictive models based on RNA features, cell type proportions, and their combination, we found that cell type composition plays a limited role in disease classification, although a subset of the observed effects may still be partially attributable to compositional shifts.

In this study, based on a comprehensive analysis of WBT data, we reported several notable findings. We identified an age-associated gene set consisting of 32 genes with strong predictive power for chronological age. This gene set includes *CD248*, which encodes a transmembrane glycoprotein involved in tissue development and inflammatory processes^31^, as well as *BACH2* and *CCR7*, which play important roles in T-cell function^32,33^, and *SATB1* and *LEF1*, which have been implicated in cellular senescence^34,35^. The functional roles of these genes warrant further investigation. Besides, our cohort included longevity individuals, which enabled us to investigate age-associated gene signatures further. We observed a significant decline in immune activity, supported by a reduced abundance of immune cell-specific gene co-expression clusters (C5 and C 16), RNA-protein regulation modules (M6 and M10). Additionally, metabolic activity was also reduced, as indicated by downregulation of metabolism-related gene co-expression clusters (C4) and flux balance analysis results.

Among the 98 health conditions analyzed, sepsis and COPD exhibited gene expression profiles that were distinctly different from those of all other conditions. In our analysis, both sepsis and COPD exhibited reduced abundance of B cell-, T cell-, mast cell- and NK cell-related gene co-expression clusters, indicating a state of immune suppression. Consistent with our findings, although sepsis and COPD differ in clinical presentation and underlying pathogenesis, they share common mechanistic features involving immune dysfunction and metabolic exhaustion. Sepsis represents a state of acute systemic inflammation. Following the cytokine storm, the disease rapidly progresses to profound immune paralysis, characterized by widespread loss of CD4⁺ and CD8⁺ T cells, a hallmark of the immunoparalysis phase^36^. In contrast, COPD is a chronic inflammatory condition that also leads to immune dysfunction and progressive T-cell exhaustion over time^37^. Consistent with our findings of diminished metabolic activity in both COPD and sepsis, previous studies have shown that both diseases are characterized by impaired energy metabolism and dysregulated lipid metabolism^38,39^. To summarize, the WBT Atlas served as an informative resource from which we effectively retrieved disease-specific signatures and gained valuable insights into the underlying mechanisms of various diseases.

We also developed a multi-class classification model using a minimal set of key RNAs with strong predictive power. For example, in psychiatric disorders, we identified RNAs highly associated with neuronal signaling and synaptic function, such as *NR4A3*, *SV2A* in bipolar disorder, *EEF1A2* in Schizophrenia. In oncology, we detected several well-characterized tumor-associated markers, such as *PTP4A2* in lung cancer, which plays a critical role in tumor progression and metastasis^40^, and *CXCL12* in stomach cancer, a key regulator of the tumor microenvironment and metastatic spread^41^. We also observed elevated *HLA-DRA* expression in breast cancer, reflecting an immune-inflamed tumor microenvironment^42^. For metabolic diseases, we identified *CREB3L3* in nonalcoholic steatohepatitis, a transcription factor essential for hepatic lipid metabolism^43^. We also identified *FGF23* in coronary artery disease, a key regulator of phosphate and vitamin D homeostasis and a well-established biomarker of cardiovascular risk^44^. Interestingly, although the RNA and protein signatures involve distinct molecules, they converge on the same pathological pathways yet function in complementary ways. This complementarity indicates that the RNA signature tends to represent intracellular regulation or the initial cellular state, while protein markers are more likely to represent the final effectors or secreted products. For instance, in sepsis, the protein markers IL1RL1, IL15, PTX3, CSF3 reflect strong inflammatory activation. Conversely, the RNA markers (e.g., *PPARG*, *CYP1B1*, *OLAH*) capture upstream immune regulation and metabolic stress, providing mutually supporting evidence for the systemic immune–metabolic collapse characteristic of sepsis^45^. Similarly, in rheumatoid arthritis, protein markers PADI4, C1QTNF1/6, CXCL1 highlight features of autoimmunity, inflammatory mediators, and cytokines. In a complementary fashion, the RNA markers *KLF15* and *GPR15* function as a transcription regulator and a chemokine receptor, respectively, which are key components associated with the immune cell differentiation and migration. We also observed that certain diseases, such as gallbladder stones and dystonia, exhibited lower performance in the multi-omics models. This may be attributable to weaker systemic alterations detectable in WBT data, as well as relatively smaller sample sizes compared to other disease cohorts.

Our study presents an unselected, population-based cohort of 4444 participants spanning 98 health conditions. Diseases with lower hospitalization rates in the general population are naturally underrepresented; thus, the observed class imbalance reflects intrinsic epidemiological characteristics. To ensure the robustness of our statistical inferences, our analysis employed established methodologies suitable for imbalanced groups. For DEG analysis, DESeq2 utilizes a negative binomial model with size-factor normalization and dispersion shrinkage. Group-level comparisons of cell-type deconvolution and metabolic activity estimation were primarily conducted using non-parametric tests (e.g., Wilcoxon), followed by FDR correction, which further mitigates potential biases introduced by varying sample sizes. Furthermore, we systematically assessed the relationship between sample size and the number of significant signals detected in this analysis (Supplementary Fig.7). No significant linear association was observed. Notably, certain diseases with smaller sample sizes, such as anaphylaxis and atopic dermatitis, still exhibited a substantial number of significant patterns, indicating that meaningful biological information can be reliably captured even within limited sample subgroups. Nevertheless, larger sample sizes remain essential for refining these estimates, and large-scale population efforts such as the Global 1 M Phenome Initiative (https://mphenome.org/) are currently underway to address this need. In addition, given the complex influence of environmental and lifestyle factors, future studies should incorporate additional confounders. Moreover, our analyses were conducted at the disease level; therefore, intra-disease stratification and pathogen-specific variability warrant further investigation.

In conclusion, we systematically characterized demographic and disease-specific gene expression features in the WBT Atlas. By integrating multiple analytical approaches with plasma proteomics data, we revealed coordinated immune and metabolic alterations as well as shifts in cell-type composition across health conditions. Moreover, leveraging condition-specific signatures, we developed a multi-class disease classification framework. Together, these findings provide new insights into systemic disease mechanisms and highlight potential diagnostic and therapeutic markers for future research.

## Methods

### Cohort description

Ethics approval for all human samples and data collection was granted by the Ethical Committee for Clinical Research of the Medical Faculty at Atatürk University, Erzurum, Turkiye (Approval No: 45, dated 30.12.2021). Informed consent was obtained before sample collection. The cohort was established by recruiting participants from January 2022 to September 2025 at Atatürk University Health Research and Application Center and Erzurum Regional Training and Research Hospital, Erzurum, Turkiye. Disease diagnoses were made according to clinical guidelines and the International Classification of Diseases, 10th Revision (ICD-10). All samples included in this study represent biologically independent individuals. No technical or biological replicates from the same subject were included. Both hospitals followed identical standard operating procedures for sample collection, processing, and storage to ensure technical and preanalytical consistency across sites. In total, 4444 participants were included in the WBT atlas, with 1280 samples having matched transcriptomic and proteomic data.

### RNA isolation and library preparation for WBT sequencing

For each participant, 2–3 ml of whole blood was collected using PAXgene tubes (BD Biosciences), which contain RNA-stabilizing reagents to preserve RNA integrity. RNA was isolated using the PAXgene QIAsymphony Blood RNA Kit (Qiagen, Hilden, Germany) on the automated extraction platform QIAsymphony. The quantity and quality of the total RNA were assessed spectrophotometrically by using a NanoDrop instrument (Thermo Fisher, USA), and the concentrations were further quantified fluorometrically by using either Qubit (Thermo Fisher) or the Victor Nivo plate reader (Perkin Elmer). RNA integrity is measured using the Agilent TapeStation system, and only samples with an RNA Integrity Number (RIN) of 5 or higher were advanced for library preparation. Transcriptomics libraries were constructed with Illumina Stranded Total RNA Prep, Ligation with Ribo-Zero, which enables the depletion of abundant RNA molecules, rRNA, and human globin mRNA. A total of 100 ng of high-quality RNA was used per sample for library construction. After verifying library fragment size and concentration, libraries were diluted to a final loading concentration of 0.4 nM. Paired-end sequencing (200 cycles) was performed on the NovaSeq 6000 platform at SZA Omics Labs (Istanbul, Turkey). The resulting raw BCL files were converted to FASTQ format and demultiplexed using DRAGEN (v4.2.7).

### Whole blood transcriptomic profiling

Transcript levels were quantified using Kallisto (v0.48.0)^46^ by pseudo-aligning reads to the human reference genome (GRCh38 cDNA). Transcript abundances were then summarized to the gene level using tximport (v1.30.0)^47^. Samples with missing metadata or insufficient sequencing quality were excluded before analysis based on pre-established quality control criteria. Specifically, samples with a pseudoalignment rate below 10% or fewer than one million processed reads were removed. These thresholds were applied to ensure sufficient and consistent sequencing quality across the dataset. Using a single-cell RNA-seq dataset of erythrocytes (GSE184916)^48^, we identified three hemoglobin-related genes: *HBB, HBA2*, and *HBA1*. To evaluate the effectiveness of globin depletion, we quantified the expression of these genes across all samples. Globin transcripts accounted for less than 0.85% of total expression, confirming the successful removal of globin RNA during the generation of transcriptomics data.

### Plasma proteomic profiling

Blood samples were collected in EDTA tubes and centrifuged at 3000 rpm for 10 min at 4 °C to separate plasma from cellular components. The isolated plasma was then aliquoted and stored at −80 °C until proteomics analysis. The plasma proteomics profiles were measured at the SciLifeLab Affinity Proteomics and National Genomics Infrastructure (NGI) in Uppsala, using the Olink Explore HT platform. In short, the proximity extension assay (PEA) employs pairs of antibodies conjugated to complementary DNA probes, enabling target-specific binding, followed by PCR amplification and detection via next-generation sequencing. Protein abundances are expressed as normalized protein expression (NPX) values on a log2 scale. Data normalization was conducted by the provider using their intensity-based normalization procedure. Quality control (QC) procedures included removal of assays flagged with QC warnings in more than 50% of all measured samples, as well as samples with QC warnings in more than 50% of all measured assays. Additionally, individual measurements with QC warnings were excluded from the final dataset. Missing values accounted for less than 0.1% of all protein measurements and were imputed using the median NPX value across samples. Proteins from dilution block 8, corresponding to the most abundant proteins (*n* = 68, dilution 1:100,000), were excluded from the analyses due to technical issues following the recommendation from the provider. Outlier detection was performed using dimensionality reduction methods, including Principal Component Analysis (PCA) and Uniform Manifold Approximation and Projection (UMAP), and samples exhibiting substantial deviation from the main dataset were removed.

### Differential expression analysis

Differential expression analysis of RNA-seq data was performed using the DESeq2 R package (v1.36.0)^49^, with adjustments for sex and age effects. To evaluate the effect of age, we compared two models for each gene: a linear model including age and sex, and a quadratic model including age, age², and sex. An ANOVA was used to test whether the inclusion of the age² term significantly improved model fit. *P* values were adjusted for multiple testing using the Benjamini–Hochberg procedure, and genes with an FDR-corrected *p* value below 0.05 were considered to exhibit a significant non-linear association with age. Our analysis showed that more than half of the genes displayed non-linear age effects. To avoid multicollinearity, we included scale(age) and scale(age)² as covariates in the differential expression analysis. Significantly differentially expressed genes (DEGs) were identified using a threshold of adjusted *p* value < 0.05. For proteomics data, differential expression analysis was conducted using the Limma package (v3.58.1)^50^ with the same covariate adjustments. Significantly differentially expressed proteins (DEPs) were identified using an adjusted *p* value < 0.05.

### Gene expression profiles

Disease-specific genes were classified according to the Human Protein Atlas (HPA) criteria (see Supplementary Data 2 for details). To evaluate the effects of age, sex, and disease on gene expression variance, ANOVA models were fitted for the top 500 most variable genes using age, sex, and disease condition as independent variables. The effect size of each factor was quantified using Eta-squared (η²) with the R package effectsize (v1.0.0)^51^. Hierarchical clustering of different diseases was performed based on pairwise distances derived from Spearman’s correlation coefficients, calculated using the mean expression values of the top 1000 most variable genes across diseases. The distance metric was defined as (1–Spearman’s ρ).

### Aging genes and transcriptome-based age prediction

Aging genes were defined as the intersection between top 50 genes for which age explained the most variance and those identified in Peters et al. study^12^. This filtering resulted in a set of 32 genes. To explore the biological relevance of these age-associated genes, over-representation analysis (ORA) was performed using the Enrichr package (v3.4)^52^, which evaluates gene set enrichment against the Molecular Signatures Database (MSigDB)^13^ using all protein-coding genes as the background.

For the development of the aging prediction model, we used the 32 identified aging associated genes as features and trained regression models using data from 4444 WBT participants. Several machine-learning regression algorithms—including linear regression, ridge regression, lasso regression, random forest, XGBoost, LightGBM, and support vector regression (RBF kernel)—were evaluated using 5-fold cross-validation on the training data, with mean R² used as the optimization metric. After cross-validation and hyperparameter tuning, LightGBM emerged as the optimal model. The final optimized model was then applied to an independent testing dataset consisting of 100 healthy individuals (GSE134080^14^). To benchmark the performance of our 32-aging associated gene signature, we also trained models using the 33 aging associated genes reported in the Korean Clock Project^15^, following the same pipeline and using the same datasets.

### Gene co-expression network analysis

Weighted gene co-expression network analysis (WGCNA)^24^ was performed to identify clusters of highly correlated genes among 4444 participants, using the top 10,000 most variable genes. A signed adjacency matrix was constructed based on pairwise gene correlations, applying a soft-thresholding power of 12 to achieve scale-free topology. Hierarchical clustering was then performed on the topological overlap matrix (TOM) derived from the adjacency matrix, using a minimum module size of 30. This led to the identification of 21 distinct gene co-expression clusters.

### WBT—proteomics correlation analysis

Pearson correlations were calculated between 19,314 genes in WBT data and 5348 genes in plasma proteomics data. RNA–protein pairs with correlation coefficients (r) > 0.45 were selected to construct the RNA-protein module network. The network layout was generated using the Fruchterman–Reingold algorithm and visualized with the igraph package (v2.1.4). The Louvain algorithm was applied to cluster the network into 12 modules.

### Single-cell data analysis

To further examine the cell-type specificity of the selected gene cluster and RNA-protein modules, we utilized a publicly available PBMC dataset from 10x Genomics (PBMC 3k), which includes ~2700 peripheral blood mononuclear cells from a healthy donor (available at: https://www.10xgenomics.com/datasets). The data were processed using the Seurat package (v5.2.1)^53^. Cells with fewer than 200 or more than 2500 detected genes, or with >5% mitochondrial gene expression, were filtered out. Gene expression data were normalized using global scaling normalization and subsequently scaled before performing principal component analysis (PCA). The top 10 principal components, selected based on the Elbow plot to capture the majority of the actual biological signal, were used for cell clustering via the Louvain algorithm with a resolution parameter of 0.5. This resulted in eight distinct cell clusters. Cell types for each cluster were manually annotated according to the expression of canonical marker genes (Supplementary Data 3). Enrichment scores of gene clusters and gene–protein modules were computed using UCell^54^, a robust rank-based method that quantifies the enrichment of a gene signature within each individual cell.

### Pathway enrichment analysis

Over-representation analysis (ORA) was performed using the Enrichr package (v3.4)^52^, which evaluates gene set overlap based on curated databases. To identify biological processes significantly associated with WGCNA gene clusters and RNA-protein modules, ORA was performed using the Gene Ontology (GO) database^55,56^. Gene Set Enrichment Analysis (GSEA) was conducted using the ClusterProfiler package (v4.10.1)^57^ to assess the enrichment of gene sets across a ranked list of genes ordered by log₂ fold change from differential expression analysis. For disease-specific genes, ORA was performed on the MSigDB dataset using all protein-coding genes as the background.

### Cell deconvolution with CIBERSORTx

The deconvolution of cell types was performed using CIBERSORTx^58^, a machine learning-based framework that infers the abundances of different cell types in bulk transcriptomics data based on a signature matrix derived from a reference dataset. Two peripheral blood immune cell bulk RNA-seq datasets were used as reference profiles.

For the HPA dataset^16^, transcriptomics data were downloaded from the Human Protein Atlas (https://www.proteinatlas.org/), comprising bulk transcriptomics data for 18 immune cell types, including: MAIT T-cell, NK-cell, T-reg, basophil, classical monocyte, eosinophil, γδ T-cell (gdT-cell), intermediate monocyte, memory B-cell, memory CD4 T-cell, memory CD8 T-cell, myeloid dendritic cell (myeloid DC), naïve B-cell, naïve CD4 T-cell, naïve CD8 T-cell, neutrophil, non-classical monocyte, and plasmacytoid dendritic cell (plasmacytoid DC).

For the Monaco dataset^17^, SRA files for 127 sorted immune cell populations were downloaded from GEO (GSE107011) using the SRA Toolkit (v2.11.3) and converted into raw FASTQ files. The bulk transcriptomics data were processed using the same pipeline as our internal dataset. Based on previous reports, these samples were consolidated into 17 immune cell types, representing the most robust classification scheme for this dataset. These included: B Memory, B Naive, Basophils LD, MAIT, Monocytes C, Monocytes NC + I, NK, Neutrophils LD, Plasmablasts, T CD4 Memory, T CD4 Naive, T CD8 Memory, T CD8 Naive, T γδ Vδ2, T γδ non-Vδ2, mDCs, and pDCs.

CIBERSORTx was executed in command-line mode via Docker, with batch correction enabled using B-mode. We performed CIBERSORTx with 500 permutations, comparing the observed gene expression profiles of each sample with reconstructed profiles generated from randomly permuted data. Samples with a *p*-value < 0.05 were considered to have sufficiently reliable estimates and were retained for downstream analysis. This resulted in a total of 4444 samples using the HPA reference and 4442 samples using the Monaco dataset. To assess whether immune cell abundance varied with age while accounting for disease status, we fitted a linear regression model for each cell type:

1 $${{\rm{proportio}}}{{{\rm{n}}}}_{{{\rm{i}}}}={{{\rm{\beta }}}}_{0}+{{{\rm{\beta }}}}_{1}{{\rm{ag}}}{{{\rm{e}}}}_{{{\rm{i}}}}+{{{\rm{\beta }}}}_{2}{{\rm{conditio}}}{{{\rm{n}}}}_{{{\rm{i}}}}+{{{\rm{\epsilon }}}}_{{{\rm{i}}}}$$

For each model, we extracted the coefficient associated with age and its corresponding *P* value. Multiple testing correction across cell types was performed using the Benjamini–Hochberg false discovery rate (FDR). Cell types with FDR-adjusted *P*  <  0.05 were considered significantly associated with age.

### Genome-scale metabolic modeling

COMPASS, a flux balance analysis (FBA)-based algorithm, was used to characterize the metabolic activity across different disease conditions^20^. The reference human metabolic model was derived from Recon3D, obtained from the BiGG Models platform^59^. Reactions associated with the Miscellaneous, Transport, and Extracellular Exchange subsystems were removed before downstream analysis.

### T cell receptor/B cell receptor analysis

TCR and BCR clonotypes were extracted from raw FASTQ files using MiXCR (v4.7.0)^60^ with default settings. Clonotype fractions were calculated based on the total read count of each unique CDR3 amino acid sequence. Repertoire diversity was assessed by computing the Shannon entropy and clonality index^61^. A total of 4398 samples were successfully evaluated and included in the downstream analysis.

### Classification using machine learning

Machine learning analyses were performed using the scikit-learn package (v1.3.0)^62^ in Python (v3.11.4). We first evaluated the disease-classification power of WBT data by selecting diseases represented by more than 40 samples, resulting in a dataset of 2970 participants across 29 health conditions. This dataset was stratified by disease and split into 70% training and 30% testing subsets to preserve class distribution. Using this data, we developed both binary models (each disease versus healthy controls) and a multi-class model.

Next, to compare the predictive performance of transcriptomic and proteomic features, we analyzed 1280 participants with matched RNA–protein measurements across 28 health conditions using the same stratified 70/30 split and identical machine-learning pipelines.

Finally, we assessed the generalizability of the identified disease-specific RNA and protein signatures by testing them in independent external transcriptomic and proteomic cohorts. As a benchmark, the same machine-learning pipeline was applied using all RNA or all protein features.

Data splitting was performed prior to all preprocessing steps. Feature selection, model selection, and hyperparameter tuning were applied exclusively to the training set. The test set was held out and used solely for final evaluation, ensuring that no information from the test set influenced any stage of feature selection or model fitting. Detailed methods are described below.

### Binary model

To address class imbalance, the training dataset was resampled using SMOTE from the imbalanced-learn package (v0.12.3)^63^, resulting in a balanced class distribution for model development. For data preprocessing, both the training and testing datasets were preprocessed using the same pipeline, including median imputation fitted on the training data.

Feature selection was performed on the training dataset using SHAP (v0.46.0)^64^ in combination with XGBoost (v2.1.2)^65^. For each disease, the top signature features were ranked based on their absolute SHAP importance.

A panel of machine-learning algorithms—including K-Nearest Neighbors (KNN), Random Forest, Logistic Regression, Extra Trees, AdaBoost, XGBoost, Support Vector Machine (SVM, polynomial kernel), and Multi-Layer Perceptron (MLP)—was evaluated using stratified 5-fold cross-validation implemented via the *StratifiedKFold* function in *scikit-learn* on the training dataset. Mean accuracy across folds was used to select the best-performing model.

The selected model was then retrained on the full training dataset and applied to the testing dataset (the best model for each disease is listed in Supplementary Data 6). For each disease, four models were constructed using different feature sets: the top 10 SHAP-ranked features, top 50, top 100, and all available features.

### Multi-class model

For multi-class disease classification, we applied the same standardized machine-learning framework, which integrates preprocessing, imputation, feature scaling, and SMOTE oversampling (applied only to the training folds) into a single pipeline to prevent data leakage during cross-validation.

Feature selection followed the same SHAP-based approach as in the binary models. For each disease, the top 10 SHAP-ranked features (disease versus all remaining samples) were combined to form the multi-class feature set.

A panel of machine-learning algorithms—including KNN, Decision Tree, Random Forest, Logistic Regression, Extra Trees, AdaBoost, Bagging, SVM (polynomial kernel), and MLP—was evaluated. Each algorithm was trained and assessed using stratified 10-fold cross-validation, with weighted F1 score used as the optimization metric.

The classifier with the highest mean cross-validation performance during training was selected as the final model. Random Forest was selected for RNA-based multi-class classification, whereas Logistic Regression was optimal for the protein-only and RNA–protein combined datasets. The selected models underwent hyperparameter tuning via 10-fold cross-validation on the training data, and the optimized model was then evaluated on the independent testing dataset. Class-wise results were summarized using the *classification_report* function.

### Independent validation on external datasets

#### External transcriptomic datasets

Independent transcriptomics validation was performed using ten RNA-seq cohorts covering: GSE165082 (12 Parkinson’s Disease and 14 controls)^66^, GSE154918 (51 Sepsis and 40 controls)^67^, GSE176260 (265 Sepsis and 30 controls)^68^, GSE63311 (72 Sepsis and 11 controls)^69^, GSE110487 (62 Sepsis)^70^, GSE124326 (240 Bipolar Disorder and 240 controls)^71^, GSE112594 (195 Type 2 Diabetes)^72^, GSE114192 (52 Type 2 Diabetes, 36 controls)^73^, GSE117769 (51 Rheumatoid Arthritis, 50 controls). For cohorts containing both disease and control samples, the original dataset was used as the testing set. For disease-only cohorts, samples were merged with 100 healthy individuals from GSE134080^14^ to construct a binary testing set. All expression matrices were transformed into TPM or FPKM units prior to analysis.

#### External proteomic datasets

For proteomics validation, we selected diseases with ≥50 samples in our dataset and identified corresponding participants in UKB-PPP, resulting in 9998 individuals: breast cancer (*n* = 1430), coronary artery disease (*n* = 2436), lung cancer (*n* = 433), Parkinson’s disease (*n* = 145), type 2 diabetes (*n* = 158), and healthy controls (*n* = 5387). For each disease, an equal number of healthy controls was randomly selected to form a balanced external testing dataset.

#### Signature-based validation

For signature-based validation, only the overlapping RNA or protein features between our top 10 SHAP-ranked disease signatures and each external dataset were used as model inputs, resulting in 4–10 features per disease (mean: 8.9). To mitigate potential batch effects across cohorts, we applied a column-wise rank transformation to the RNA and protein expression matrices. For each feature, expression values across all samples were ranked and normalized to the [0,1] range by dividing by the maximum rank.

2 $$\widetilde{{X}_{{ij}}}=\frac{{{\mbox{rank}}}\,{{\mbox{of}}}\,{X}_{{ij}}{{\mbox{in}}}\,{{\mbox{column}}}{j}}{n}\,$$

A panel of machine-learning algorithms—including Logistic Regression, Decision Tree, Random Forest, AdaBoost, Bagging, SVM (polynomial kernel), and XGBoost—was assessed. Each algorithm was evaluated using stratified 5-fold cross-validation on the internal training dataset, with AUC as the optimization metric. The best-performing model was then tuned using RandomizedSearchCV (50 iterations) and retrained on the full internal training dataset before being applied to each external test cohort.

#### PCA-based benchmark

To benchmark the predictive value of disease-specific signatures, PCA-based models were constructed using all RNA or all protein features. Expression matrices were standardized using *StandardScaler* and projected onto the top 10 principal components, with PCA fitted exclusively on the internal training dataset to prevent information leakage. Missing values were imputed using the same training-derived medians.

The same classifier panel, cross-validation strategy, and hyperparameter tuning procedures described above for signature-based validation were applied to the PCA-based models. After hyperparameter optimization, the tuned PCA models were evaluated on each external cohort. Performance metrics—including accuracy, balanced accuracy, weighted F1 score, and AUC—were computed, and predicted probabilities, predicted labels, and true labels were retained for downstream analyses.

### Data visualization

Figures were generated using the ggplot2(v3.5.1)^74^ package, with additional support from ggbeeswarm (v0.7.2)^75^ and ggrepel (v0.9.6)^76^. UMAP dimensionality reduction plots of single-cell datasets, based on the top 10 principal components, were created using the Seurat package (v5.2.1)^53^. Network visualizations were performed in Cytoscape^77^.

### Statistical analysis

Differences in WGCNA module eigengenes and COMPASS-derived metabolic activity between disease groups were quantified using Cohen’s d effect size, which measures the magnitude of difference relative to overall variance. It is defined as the difference in group means divided by the pooled standard deviation:

3 $${{\mbox{Cohen}}}{\mbox{'}}{{\rm{s}}}\,{d}=\frac{\bar{{x}_{1}}-\bar{{x}_{2}}}{{s}_{p}}$$

4 $${s}_{p}=\sqrt{\frac{\left({n}_{1}-1\right){s}_{1}^{2}+\left({n}_{2}-1\right){s}_{2}^{2}}{{n}_{1}+{n}_{2}-2}}$$

Here $\bar{{x}_{1}}{{\rm{and}}}\bar{{x}_{2}}$ are the group means, *s*_1_^2^and s_2_^2^ are the group variances, and *n*_1_ and *n*_2_ are the sample sizes of the two groups. Statistical significance was assessed using the Wilcoxon rank-sum test.

For CIBERSORTx and MiXCR results, differences between disease and healthy groups were evaluated using log₂ fold change, and significance was also determined using the Wilcoxon rank-sum test. *p*-values were adjusted for multiple comparisons using the Benjamini-Hochberg method^78^. All statistical analyses were done by R (v4.3.1).

### Reporting summary

Further information on research design is available in the Nature Portfolio Reporting Summary linked to this article.

## Supplementary information

Supplementary Information

Transparent Peer Review file

Reporting Summary

Description of Additional Supplementary Files

Supplementary Dataset 1

Supplementary Dataset 2

Supplementary Dataset 3

Supplementary Dataset 4

Supplementary Dataset 5

Supplementary Dataset 6

## Source data

Source Data

## Data Availability

Disease-level gene expression results are publicly available through (https://www.phenomeatlas.org/wbt/). All other data orting the findings of this study are provided within the paper, its Supplementary Information, and the Source Data. Owing to participant consent and legal restrictions, the individual-level datasets are not publicly available. Researchers seeking access for non-commercial research and validation purposes may submit a request to the corresponding author, AM (adilm@scilifelab.se). Requests will be reviewed internally within two to four weeks of submission, subject to the completeness of the application and any necessary approvals, to ensure compliance with ethical considerations, participant consent agreements, and applicable laws and regulations. Access will be provided where the proposed use is consistent with these requirements. Source data are provided with this paper.

## Code availability

All code necessary for the data analysis and visualization is available at: https://github.com/Mengzhensw/WBT_Atlas, and has been archived with a permanent DOI via Zenodo: https://doi.org/10.5281/zenodo.21512417.

## References

[1] Liston, A., Humblet-Baron, S., Duffy, D. & Goris, A. Human immune diversity: from evolution to modernity. *Nat. Immunol.* **22**, 1479–1489 10.1038/s41590-021-01058-1 (2021). 34795445

[2] Uhlén, M. et al. Proteomics. Tissue-based map of the human proteome. *Science* **347**, 1260419 10.1126/science.1260419 (2015). 25613900

[3] Sudlow, C. et al. UK Biobank: an open access resource for identifying the causes of a wide range of complex diseases of middle and old age. *PLoS Med* **12**, e1001779 10.1371/journal.pmed.1001779 (2015). PMC4380465 25826379

[4] Cancer Genome Atlas Research, N. et al. The cancer genome Atlas Pan-cancer Analysis Project. *Nat. Genet* **45**, 1113–1120 10.1038/ng.2764 (2013). PMC3919969 24071849

[5] Consortium, G. T. et al. Human genomics. The Genotype-Tissue Expression (GTEx) pilot analysis: multitissue gene regulation in humans. *Science* **348**, 648–660, 10.1126/science.1262110 (2015). PMC4547484 25954001

[6] Claussnitzer, M. et al. A brief history of human disease genetics. *Nature* **577**, 179–189 10.1038/s41586-019-1879-7 (2020). PMC7405896 31915397

[7] Deng, Y.-T. et al. Atlas of the plasma proteome in health and disease in 53,026 adults. *Cell* **188**, 253–271.e257 10.1016/j.cell.2024.10.045 (2025). 39579765

[8] Álvez, M. B. et al. Next generation pan-cancer blood proteome profiling using proximity extension assay. *Nat. Commun.* **14**, 4308 10.1038/s41467-023-39765-y (2023). PMC10354027 37463882

[9] Álvez, M. B. et al. A human pan-disease blood atlas of the circulating proteome. *Science* **390**, 2678 10.1126/science.adx2678 (2025). 41066540

[10] Kim, J. & Eberwine, J. RNA: state memory and mediator of cellular phenotype. *Trends Cell Biol.* **20**, 311–318 10.1016/j.tcb.2010.03.003 (2010). PMC2892202 20382532

[11] Argmann, C. et al. Biopsy and blood-based molecular biomarker of inflammation in IBD. *Gut* **72**, 1271–1287 10.1136/gutjnl-2021-326451 (2023). PMC10014487 36109152

[12] Peters, M. J. et al. The transcriptional landscape of age in human peripheral blood. *Nat. Commun.* **6**, 8570 10.1038/ncomms9570 (2015). PMC4639797 26490707

[13] Liberzon, A. et al. The Molecular Signatures Database (MSigDB) hallmark gene set collection. *Cell Syst.* **1**, 417–425 10.1016/j.cels.2015.12.004 (2015). PMC4707969 26771021

[14] Aguirre-Gamboa, R. et al. Deconvolution of bulk blood eQTL effects into immune cell subpopulations. *BMC Bioinforma.* **21**, 243 10.1186/s12859-020-03576-5 (2020). PMC7291428 32532224

[15] An, K. et al. Dynamic and reversible transcriptomic age shifts induced by COVID-19 in Korean whole blood. *Aging (Albany, NY)* **17**, 1484–1510, 10.18632/aging.206270 (2025). PMC12245201 40500114

[16] Uhlen, M. et al. A genome-wide transcriptomic analysis of protein-coding genes in human blood cells. *Science* **366**, eaax9198 10.1126/science.aax9198 (2019). 31857451

[17] Monaco, G. et al. RNA-Seq Signatures Normalized by mRNA Abundance Allow Absolute Deconvolution of Human Immune Cell Types. *Cell Rep.* **26**, 1627–1640 10.1016/j.celrep.2019.01.041 (2019). PMC6367568 30726743

[18] Mardinoglu, A. & Nielsen, J. New paradigms for metabolic modeling of human cells. *Curr. Opin. Biotechnol.* **34**, 91–97 10.1016/j.copbio.2014.12.013 (2015). 25559199

[19] Mardinoglu, A. & Palsson, B. Ø Genome-scale models in human metabologenomics. *Nat. Rev. Genet* **26**, 123–140, 10.1038/s41576-024-00768-0 (2025). 39300314

[20] Wagner, A. et al. Metabolic modeling of single Th17 cells reveals regulators of autoimmunity. *Cell* **184**, 4168–4185 10.1016/j.cell.2021.05.045 (2021). PMC8621950 34216539

[21] Cibrian, D., de la Fuente, H. & Sánchez-Madrid, F. Metabolic pathways that control skin homeostasis and inflammation. *Trends Mol. Med* **26**, 975–986 10.1016/j.molmed.2020.04.004 (2020). 32371170

[22] Cloonan, S. M., Kim, K., Esteves, P., Trian, T. & Barnes, P. J. Mitochondrial dysfunction in lung ageing and disease. *Eur. Respir. Rev.* **29**, 200165 10.1183/16000617.0165-2020 (2020). PMC9488551 33060165

[23] Dasgupta, S., Ghosh, N., Bhattacharyya, P., Roy Chowdhury, S. & Chaudhury, K. Metabolomics of asthma, COPD, and asthma-COPD overlap: an overview. *Crit. Rev. Clin. Lab Sci.* **60**, 153–170 10.1080/10408363.2022.2140329 (2023). 36420874

[24] Langfelder, P. & Horvath, S. WGCNA: an R package for weighted correlation network analysis. *BMC Bioinforma.* **9**, 559 10.1186/1471-2105-9-559 (2008). PMC2631488 19114008

[25] Furman, D. et al. Chronic inflammation in the etiology of disease across the life span. *Nat. Med* **25**, 1822–1832 10.1038/s41591-019-0675-0 (2019). PMC7147972 31806905

[26] López-Otín, C., Blasco, M. A., Partridge, L., Serrano, M. & Kroemer, G. The hallmarks of aging. *Cell* **153**, 1194–1217 10.1016/j.cell.2013.05.039 (2013). PMC3836174 23746838

[27] O’Connell, G. C., Wang, J. & Smothers, C. Donor white blood cell differential is the single largest determinant of whole blood gene expression patterns. *Genomics* **115**, 110708 10.1016/j.ygeno.2023.110708 (2023). PMC10872590 37730167

[28] Mastrokolias, A. et al. Increased sensitivity of next generation sequencing-based expression profiling after globin reduction in human blood RNA. *BMC Genom.* **13**, 28 10.1186/1471-2164-13-28 (2012). PMC3275489 22257641

[29] Collinson, R. J. et al. PlateletSeq: a novel method for discovery of blood-based biomarkers. *Methods* **219**, 139–149 10.1016/j.ymeth.2023.10.003 (2023). 37813292

[30] Zhong, P. et al. A comprehensive review on circulating cfRNA in Plasma: implications for disease diagnosis and beyond. *Diagnostics (Basel)* **14**, 1045 10.3390/diagnostics14101045 (2024). PMC11119755 38786343

[31] Teicher, B. A. CD248: a therapeutic target in cancer and fibrotic diseases. *Oncotarget* **10**, 993–1009 10.18632/oncotarget.26590 (2019). PMC6398180 30847027

[32] Chi, V. L. D. et al. Age-related changes in the BACH2 and PRDM1 genes in lymphocytes from healthy donors and chronic lymphocytic leukemia patients. *BMC Cancer* **19**, 81 10.1186/s12885-019-5276-2 (2019). PMC6337793 30654767

[33] Da Mesquita, S. et al. Aging-associated deficit in CCR7 is linked to worsened glymphatic function, cognition, neuroinflammation, and β-amyloid pathology. *Sci. Adv.* **7**, eabe4601 10.1126/sciadv.abe4601 (2021). PMC8139596 34020948

[34] Riessland, M. et al. Loss of SATB1 induces p21-dependent cellular senescence in post-mitotic dopaminergic neurons. *Cell Stem Cell* **25**, 514–530 10.1016/j.stem.2019.08.013 (2019). PMC7493192 31543366

[35] Jia, M. et al. LEF1 isoforms regulate cellular senescence and aging. *Aging Cell* **22**, e14024 10.1111/acel.14024 (2023). PMC10726832 37961030

[36] Jensen, I. J., Sjaastad, F. V., Griffith, T. S. & Badovinac, V. P. Sepsis-induced T cell immunoparalysis: the ins and outs of impaired T cell immunity. *J. Immunol.* **200**, 1543–1553 10.4049/jimmunol.1701618 (2018). PMC5826615 29463691

[37] Hou, J. & Sun, Y. Role of regulatory T cells in disturbed immune homeostasis in patients with chronic obstructive pulmonary disease. *Front Immunol.* **11**, 723 10.3389/fimmu.2020.00723 (2020). PMC7198877 32411140

[38] Hu, D. et al. Mitochondrial dysfunction in sepsis: mechanisms and therapeutic perspectives. *Crit. Care* **28**, 292 10.1186/s13054-024-05069-w (2024). PMC11373266 39227925

[39] Liang, Q., Wang, Y. & Li, Z. Lipid metabolism reprogramming in chronic obstructive pulmonary disease. *Mol. Med* **31**, 129 10.1186/s10020-025-01191-9 (2025). PMC11974042 40197131

[40] Wei, M., Korotkov, K. V. & Blackburn, J. S. Targeting phosphatases of regenerating liver (PRLs) in cancer. *Pharm. Ther.* **190**, 128–138 10.1016/j.pharmthera.2018.05.014 (2018). PMC6192704 29859177

[41] Yang, Y. et al. CXCL12-CXCR4/CXCR7 axis in cancer: from mechanisms to clinical applications. *Int J. Biol. Sci.* **19**, 3341–3359 10.7150/ijbs.82317 (2023). PMC10367567 37497001

[42] Liu, D. & Hofman, P. Expression of NOTCH1, NOTCH4, HLA-DMA and HLA-DRA is synergistically associated with T cell exclusion, immune checkpoint blockade efficacy a nd recurrence risk in ER-negative breast cancer. *Cell Oncol. (Dordr.)* **45**, 463–477 10.1007/s13402-022-00677-6 (2022). PMC12978109 35543859

[43] Nakagawa, Y. et al. Enterohepatic transcription factor CREB3L3 protects Atherosclerosis via SREBP competitive inhibition. *Cell Mol. Gastroenterol. Hepatol.* **11**, 949–971 10.1016/j.jcmgh.2020.11.004 (2021). PMC7900604 33246135

[44] Stöhr, R., Schuh, A., Heine, G. H. & Brandenburg, V. FGF23 in cardiovascular disease: innocent bystander or active mediator? *Front. Endocrinol.* **9**, 351 10.3389/fendo.2018.00351 (2018). PMC6036253 30013515

[45] von Knethen, A., Soller, M. & Brüne, B. Peroxisome proliferator-activated receptor gamma (PPAR gamma) and sepsis. *Arch. Immunol. Ther. Exp. (Warsz.)* **55**, 19–25 10.1007/s00005-007-0005-y (2007). 17277894

[46] Bray, N. L., Pimentel, H., Melsted, P. & Pachter, L. Near-optimal probabilistic RNA-seq quantification. *Nat. Biotechnol.* **34**, 525–527 10.1038/nbt.3519 (2016). 27043002

[47] Soneson, C., Love, M. I. & Robinson, M. D. Differential analyses for RNA-seq: transcript-level estimates improve gene-level inferences. *F1000Res* **4**, 1521 10.12688/f1000research.7563.2 (2015). PMC4712774 26925227

[48] Jain, V. et al. Single cell RNA-Seq analysis of human red cells. *Front Physiol.* **13**, 828700 10.3389/fphys.2022.828700 (2022). PMC9065680 35514346

[49] Love, M. I., Huber, W. & Anders, S. Moderated estimation of fold change and dispersion for RNA-seq data with DESeq2. *Genome Biol.* **15**, 550 10.1186/s13059-014-0550-8 (2014). PMC4302049 25516281

[50] Ritchie, M. E. et al. limma powers differential expression analyses for RNA-sequencing and microarray studies. *Nucleic Acids Res* **43**, e47–e47 10.1093/nar/gkv007 (2015). PMC4402510 25605792

[51] Ben-Shachar, M. S., Lüdecke, D. & Makowski, D. effectsize: Estimation of effect size indices and standardized parameters. *J. Open Source Softw*. **5**, 2815 10.21105/joss.02815 (2020).

[52] Chen, E. Y. et al. Enrichr: interactive and collaborative HTML5 gene list enrichment analysis tool. *BMC Bioinforma.* **14**, 128 10.1186/1471-2105-14-128 (2013). PMC3637064 23586463

[53] Hao, Y. et al. Dictionary learning for integrative, multimodal and scalable single-cell analysis. *Nat. Biotechnol.* **42**, 293–304 10.1038/s41587-023-01767-y (2024). PMC10928517 37231261

[54] Andreatta, M. & Carmona, S. J. UCell: Robust and scalable single-cell gene signature scoring. *Comput. Struct. Biotechnol. J.* **19**, 3796–3798 10.1016/j.csbj.2021.06.043 (2021). PMC8271111 34285779

[55] Ashburner, M. et al. Gene ontology: tool for the unification of biology. *Nat. Genet* **25**, 25–29 10.1038/75556 (2000). PMC3037419 10802651

[56] Gene Ontology, C. et al. The Gene Ontology Knowledgebase in 2023. *Genetics* **224**, iyad031 10.1093/genetics/iyad031 (2023). PMC10158837 36866529

[57] Yu, G., Wang, L.-G., Han, Y. & He, Q.-Y. clusterProfiler: an R package for comparing biological themes among gene clusters. *OMICS*. *Omics : a J. Integr. Biol.* **16**, 284–287 10.1089/omi.2011.0118 (2012). PMC3339379 22455463

[58] Newman, A. M. et al. Determining cell type abundance and expression from bulk tissues with digital cytometry. *Nat. Biotechnol.* **37**, 773–782 10.1038/s41587-019-0114-2 (2019). PMC6610714 31061481

[59] King, Z. A. et al. BiGG Models: A platform for integrating, standardizing and sharing genome-scale models. *Nucleic Acids Res* **44**, D515–D522 10.1093/nar/gkv1049 (2016). PMC4702785 26476456

[60] Bolotin, D. A. et al. MiXCR: software for comprehensive adaptive immunity profiling. *Nat. Methods* **12**, 380–381 10.1038/nmeth.3364 (2015). 25924071

[61] Chiffelle, J. et al. T-cell repertoire analysis and metrics of diversity and clonality. *Curr. Opin. Biotechnol.* **65**, 284–295 10.1016/j.copbio.2020.07.010 (2020). 32889231

[62] Pedregosa, F. et al. Scikit-learn: machine learning in Python. *J. Mach. Learn. Res.* **12**, 2825–2830 (2011).

[63] LemaÃŽtre, G., Nogueira, F. & Aridas, C. K. Imbalanced-learn: a Python toolbox to tackle the curse of imbalanced datasets in machine learning. *J. Mach. Learn. Res.* **18**, 1–5 (2017).

[64] Lundberg, S. M. & Lee, S.-I. A unified approach to interpreting model predictions. *Adv. Neural Inf. Process. Syst.* **30**, 4768–4777 (2017).

[65] Chen, T. & Guestrin, C. XGBoost: A scalable tree boosting system. *Proc. 22nd ACM SIGKDD Int. Conf. Knowl. Discov. Data Min*. 785–794 10.1145/2939672.2939785 (2016).

[66] Henderson, A. R. et al. DNA methylation and expression profiles of whole blood in Parkinson’s disease. *Front Genet* **12**, 640266 10.3389/fgene.2021.640266 (2021). PMC8107387 33981329

[67] Herwanto, V. et al. Blood transcriptome analysis of patients with uncomplicated bacterial infection and sepsis. *BMC Res Notes* **14**, 76 10.1186/s13104-021-05488-w (2021). PMC7913415 33640018

[68] Steinbrink, J. M. et al. The host transcriptional response to Candidemia is dominated by neutrophil activation and heme biosynthesis and supports novel diagnostic approaches. *Genome Med* **13**, 108 10.1186/s13073-021-00924-9 (2021). PMC8259367 34225776

[69] Pena, O. M. et al. An endotoxin tolerance signature predicts sepsis and organ dysfunction at initial clinical presentation. *EBioMedicine* **1**, 64–71 10.1016/j.ebiom.2014.10.003 (2014). PMC4326653 25685830

[70] Barcella, M. et al. Identification of a transcriptome profile associated with improvement of organ function in septic shock patients after early supportive therapy. *Crit. Care* **22**, 312 10.1186/s13054-018-2242-3 (2018). PMC6249814 30463588

[71] Krebs, C. E. et al. Whole blood transcriptome analysis in bipolar disorder reveals strong lithium effect. *Psychol. Med* **50**, 2575–2586 10.1017/S0033291719002745 (2020). 31589133

[72] Dufort, M. J., Greenbaum, C. J., Speake, C. & Linsley, P. S. Cell type-specific immune phenotypes predict loss of insulin secretion in new-onset type 1 diabetes. *JCI Insight* **4**, e125556 10.1172/jci.insight.125556 (2019). PMC6478408 30830868

[73] Eckold, C. et al. Impact of intermediate hyperglycemia and diabetes on immune dysfunction in tuberculosis. *Clin. Infect. Dis.* **72**, 69–78, 10.1093/cid/ciaa751 (2021). PMC7823074 32533832

[74] Wickham, H. et al. in *ggplot2: Elegant Graphics for Data Analysis,* 189–201 (Springer, 2016).

[75] Clarke, E., Sherrill-Mix, S. & Dawson, C. ggbeeswarm: Categorical scatter (violin point) plots. *CRAN: Contributed Packages* (2016).

[76] Slowikowski, K. et al. Package ggrepel. *Automatically position non-overlapping text labels with ‘ggplot2* (2018).

[77] Shannon, P. et al. Cytoscape: a software environment for integrated models of biomolecular interaction networks. *Genome Res* **13**, 2498–2504 10.1101/gr.1239303 (2003). PMC403769 14597658

[78] Benjamini, Y. & Hochberg, Y. Controlling the false discovery rate: a practical and powerful approach to multiple testing. *J. R. Stat. Soc.: Ser. B (Methodol.)* **57**, 289–300 (1995).

