# Supplementary material for: Whole blood transcriptomics atlas across human diseases
Source: Nat Commun. 2026 Oct 12;17:11547. doi: 10.1038/s41467-026-78440-w (PMC13748637; doi:10.1038/s41467-026-78440-w)
Supplement: Supplementary file 1 [file 41467_2026_78440_MOESM1_ESM.pdf]

Whole Blood Transcriptomics Atlas Across Human Diseases

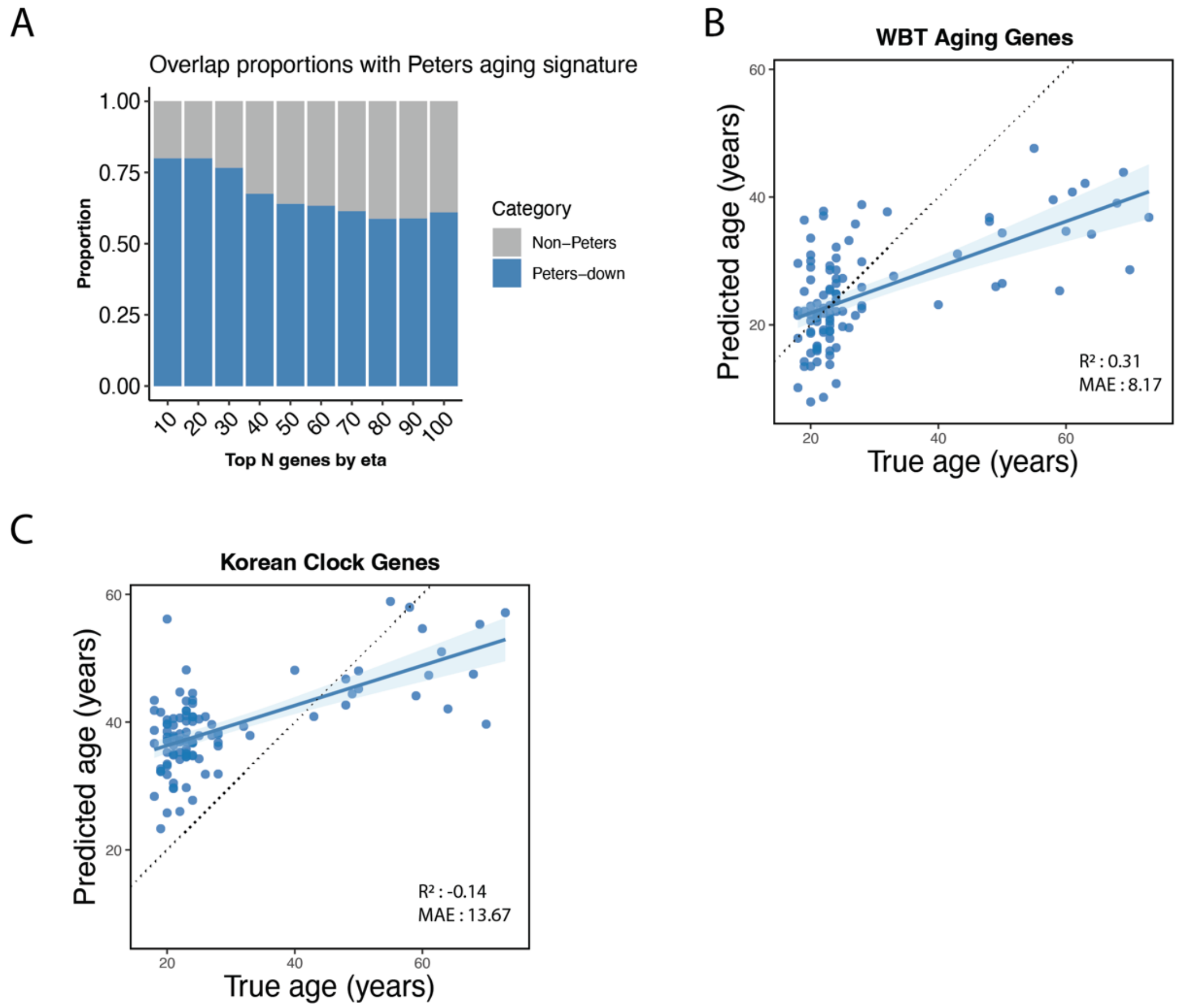

Supplementary Fig. 1

(A) Overlap between the top aging-related genes identified in our study and the aging signatures reported by Peter et al. (DOI: 10.1038/ncomms9570) .

(B) Aging prediction model based on the 32 aging-related genes identified in our study.

(C) Aging prediction model based on the gene set from the Korean Clock Project.

Source data are provided in the Source data file.

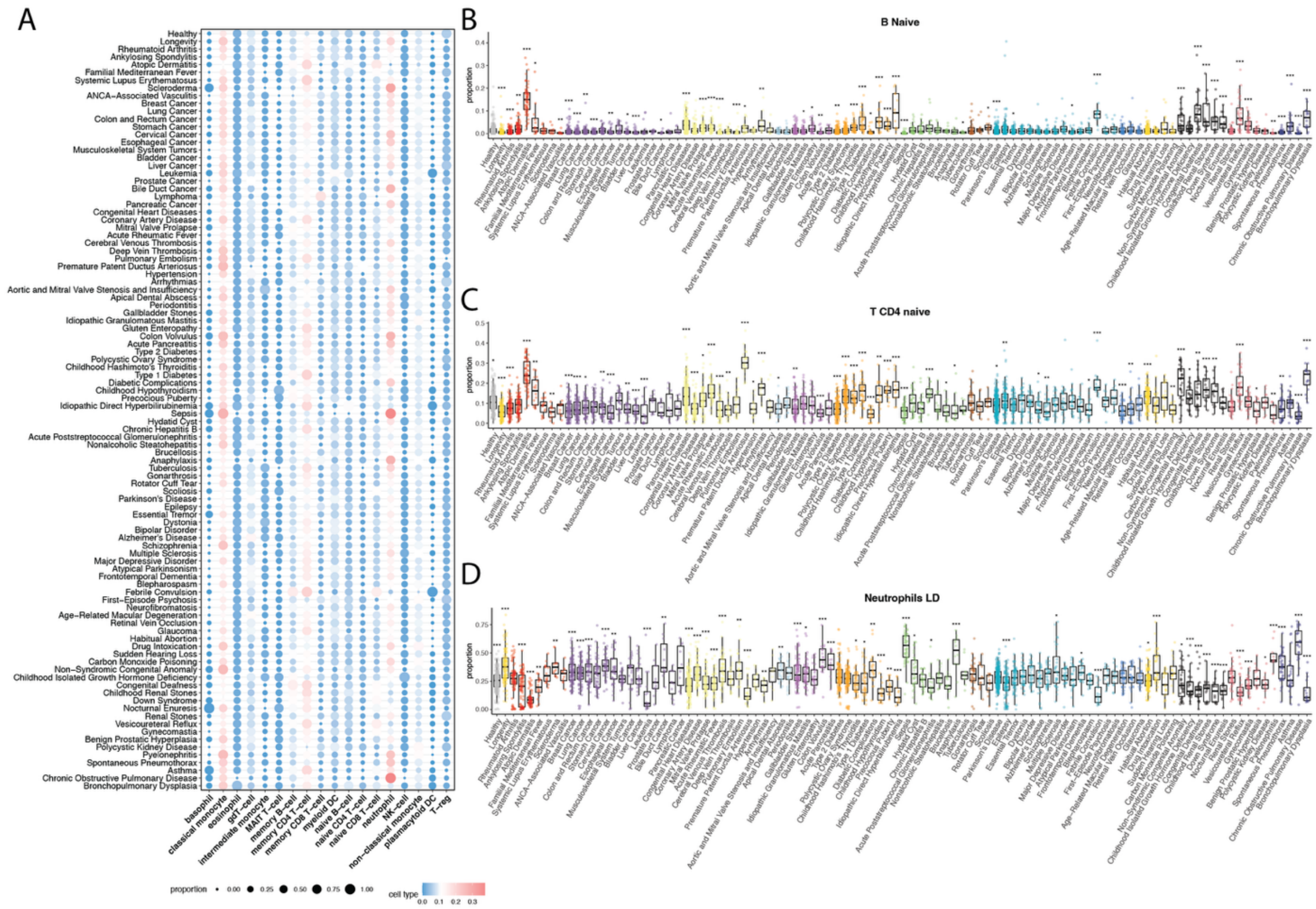

Supplementary Fig. 2

(A) Bubble plot showing the mean immune cell proportions inferred by CIBERSORTx using the HPA reference. Bubble color represents the percentage of each immune cell type within a given disease, while bubble size indicates the normalized cell fraction across diseases, enabling cross-condition comparison.

(B–D) Relative proportions of B naive cells, T CD4 naive cells and neutrophils across multiple diseases, estimated using the Monaco reference. Each disease was compared with all others using a two-sided Wilcoxon rank-sum test, and p values were adjusted for multiple comparisons using the Benjamini–Hochberg (BH) method. Box limits indicate the interquartile range (25th–75th percentile), the center line indicates the median, and whiskers extend to the most extreme data point within 1.5× the interquartile range. Significance levels: *** $p < 0.001$, ** $0.001 < p < 0.01$, * $0.01 < p < 0.05$. Sample number and exact p-value in source data file.

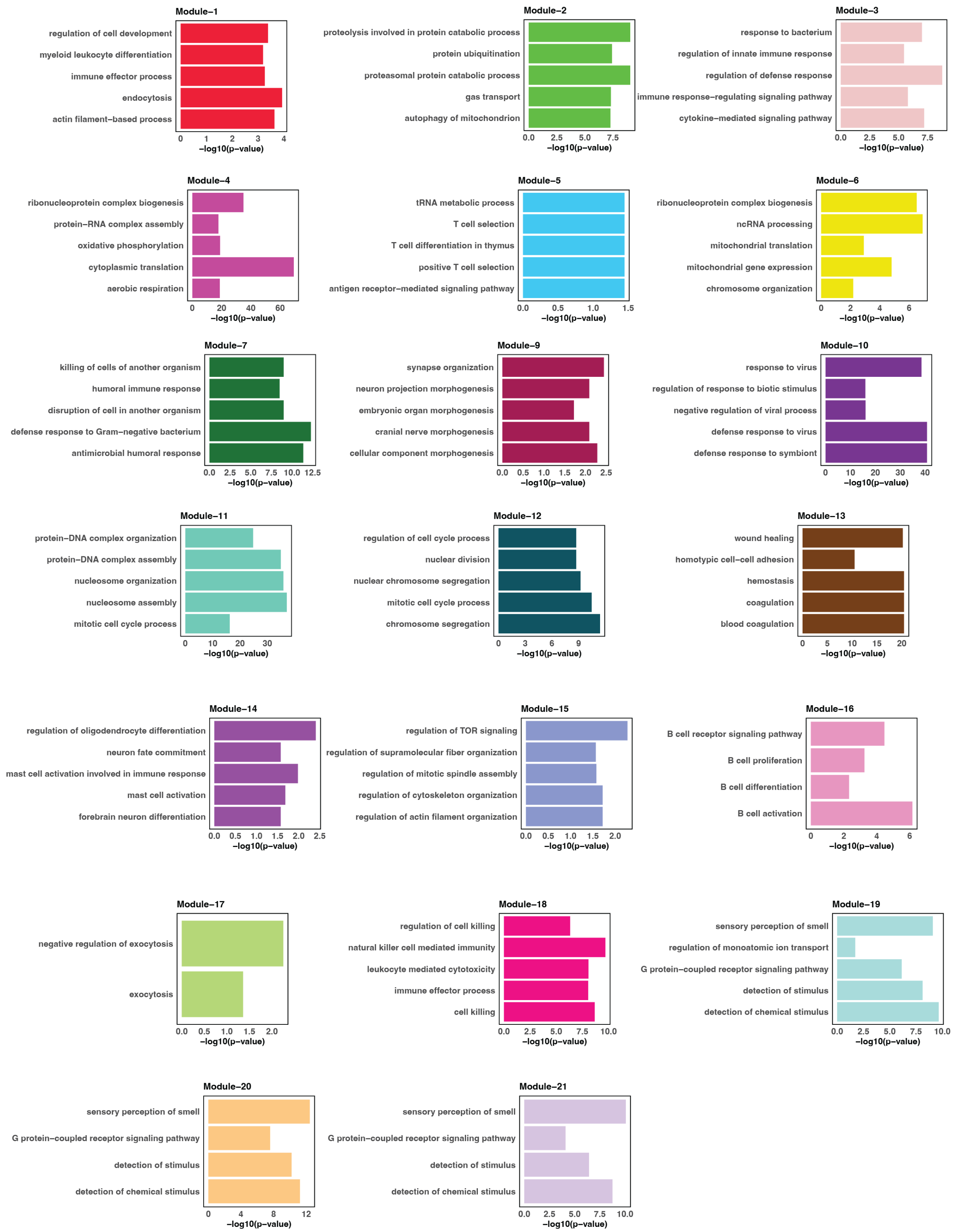

Supplementary Fig. 3

Top enriched Gene Ontology (GO) pathways of different Weighted Gene Co-expression Network Analysis (WGCNA) modules.

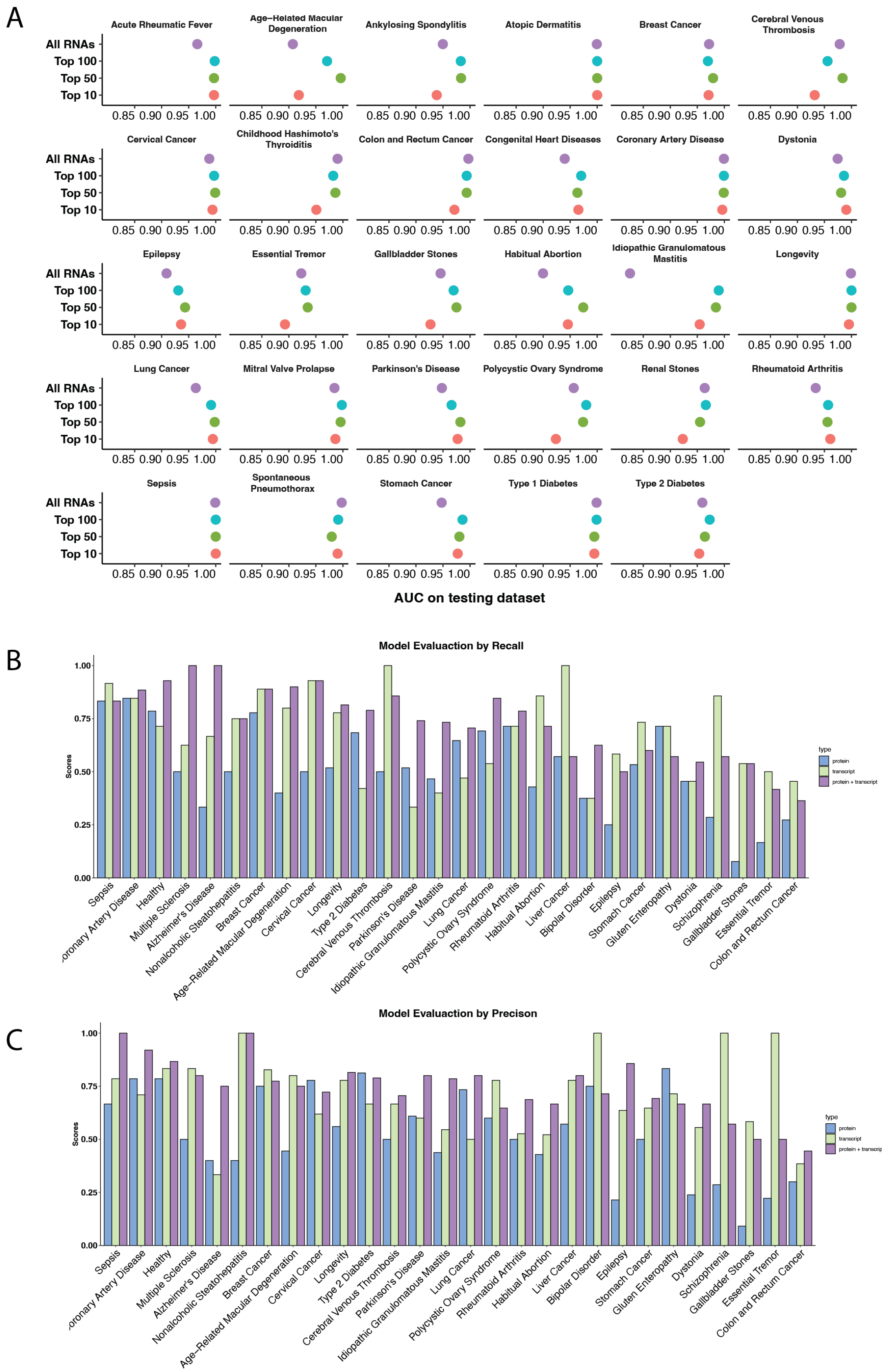
A
Acute Rheumatic Fever
Age−Related Macular Degeneration
Ankylosing Spondylitis
Atopic Dermatitis
Breast Cancer
Cerebral Venous Thrombosis
Cervical Cancer
Childhood Hashimoto's Thyroiditis
Colon and Rectum Cancer
Congenital Heart Diseases
Coronary Artery Disease
Dystonia
Epilepsy
Essential Tremor
Gallbladder Stones
Habitual Abortion
Idiopathic Granulomatous Mastitis
Longevity
Lung Cancer
Mitral Valve Prolapse
Parkinson's Disease
Polycystic Ovary Syndrome
Renal Stones
Rheumatoid Arthritis
Sepsis
Spontaneous Pneumothorax
Stomach Cancer
Type 1 Diabetes
Type 2 Diabetes
All RNAs
Top 100
Top 50
Top 10
0.85 0.90 0.95 1.00
AUC on testing dataset
B
Model Evaluaction by Recall
Scores
0.00
0.25
0.50
0.75
1.00
type
protein
transcript
protein + transcript
Sepsis
Coronary Artery Disease
Healthy
Multiple Sclerosis
Alzheimer's Disease
Nonalcoholic Steatohepatitis
Breast Cancer
Age−Related Macular Degeneration
Cervical Cancer
Longevity
Type 2 Diabetes
Cerebral Venous Thrombosis
Parkinson's Disease
Idiopathic Granulomatous Mastitis
Lung Cancer
Polycystic Ovary Syndrome
Rheumatoid Arthritis
Habitual Abortion
Liver Cancer
Bipolar Disorder
Epilepsy
Stomach Cancer
Gluten Enteropathy
Dystonia
Schizophrenia
Gallbladder Stones
Essential Tremor
Colon and Rectum Cancer
C
Model Evaluaction by Precison
Scores
0.00
0.25
0.50
0.75
1.00
type
protein
transcript
protein + transcript

Supplementary Fig.4

(A) Binary-classification performance of RNA features in distinguishing each disease from healthy controls across 29 health conditions (sample size > 40). For each condition, four feature sets were evaluated—including the top 10, top 50, and top 100 SHAP-ranked features, as well as all features. Notably, the top 10 features maintained strong predictive performance, comparable to substantially larger feature sets.

(B–C) Recall and precision of RNA, protein, and combined RNA–protein data in a multi-class model distinguishing 28 health conditions using transcriptomic–proteomic–matched samples and a consistent training pipeline. The feature sets included the top 10 SHAP-ranked RNA features for the RNA model, the top 10 SHAP-ranked protein features for the protein model, and the combined top 10 RNA plus top 10 protein features for the integrated model.

Source data are provided in the Source data file.

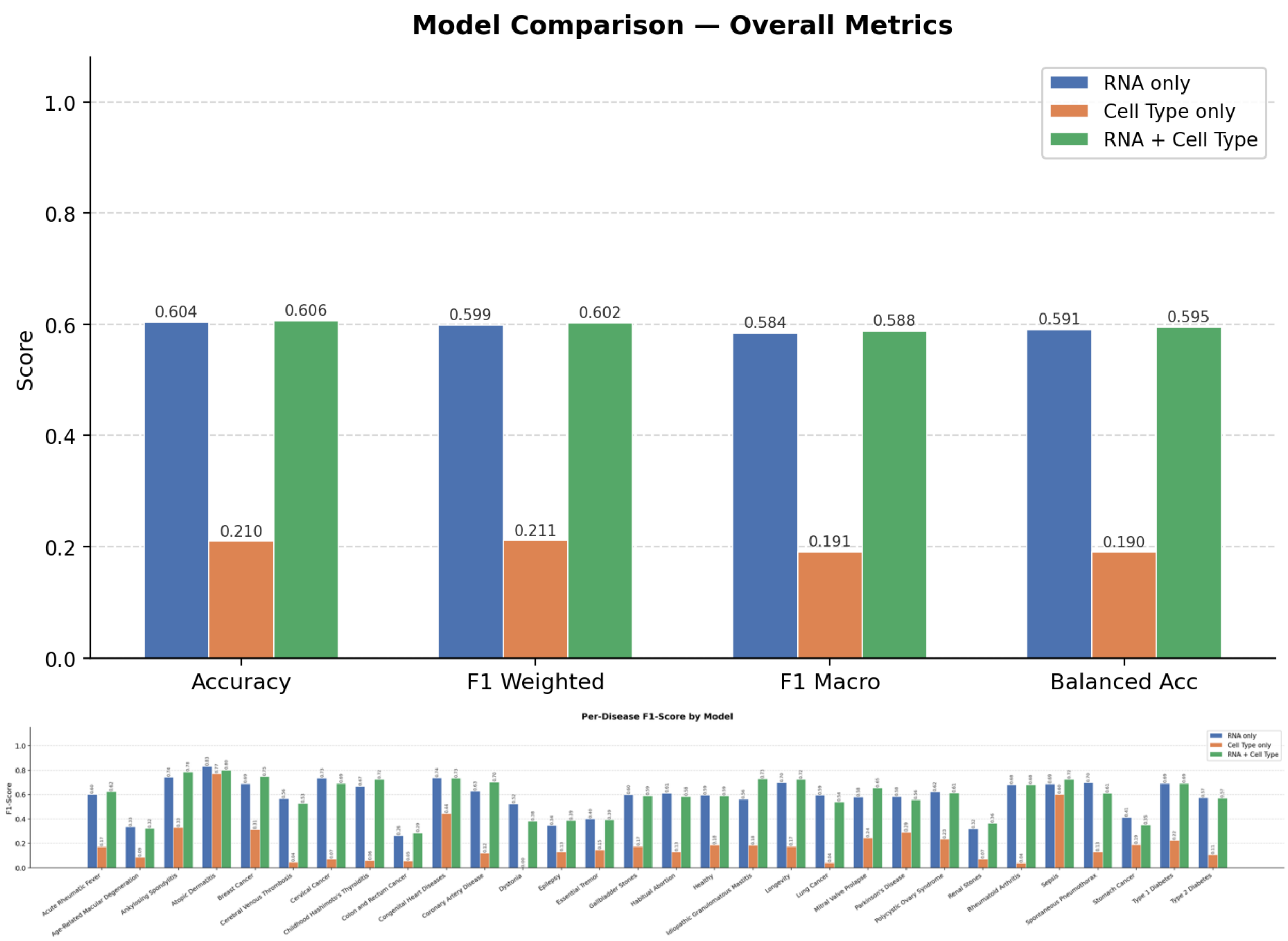

Supplementary Fig. 5

(A) Overall multi-class classification performance (accuracy, weighted F1, macro F1, and balanced accuracy) across 29 disease conditions, comparing three feature sets: RNA only (SHAP top 10 genes), cell type proportions only, and their combination.

(B) Disease-specific F1 scores for each of the 29 conditions using the same three feature sets. Source data are provided in the Source data file.

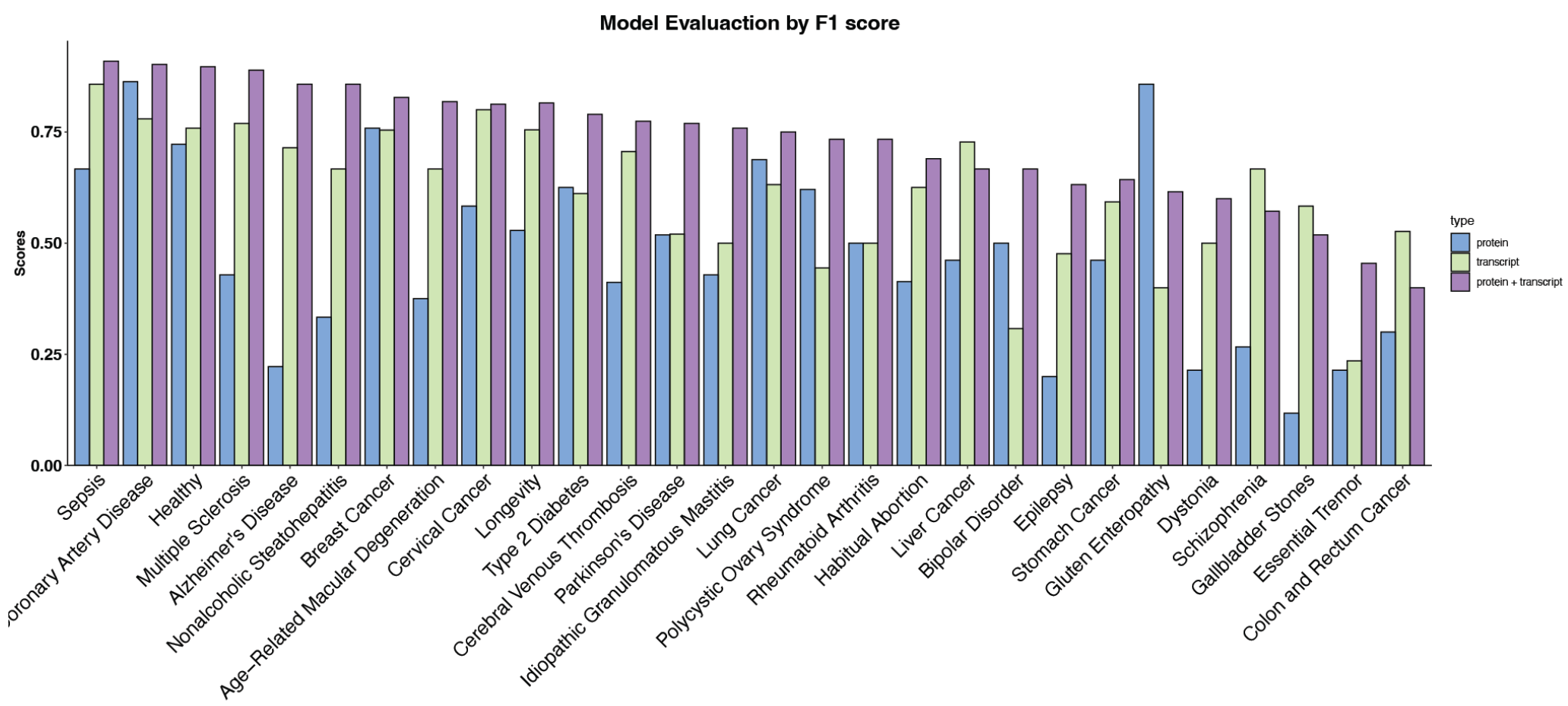
Model Evaluaction by F1 score
Scores
0.00
0.25
0.50
0.75
type
protein
transcript
protein + transcript
Sepsis
oronary Artery Disease
Healthy
Multiple Sclerosis
Alzheimer's Disease
Nonalcoholic Steatohepatitis
Breast Cancer
Age−Related Macular Degeneration
Cervical Cancer
Longevity
Type 2 Diabetes
Cerebral Venous Thrombosis
Parkinson's Disease
Idiopathic Granulomatous Mastitis
Lung Cancer
Polycystic Ovary Syndrome
Rheumatoid Arthritis
Habitual Abortion
Liver Cancer
Bipolar Disorder
Epilepsy
Stomach Cancer
Gluten Enteropathy
Dystonia
Schizophrenia
Gallbladder Stones
Essential Tremor
Colon and Rectum Cancer

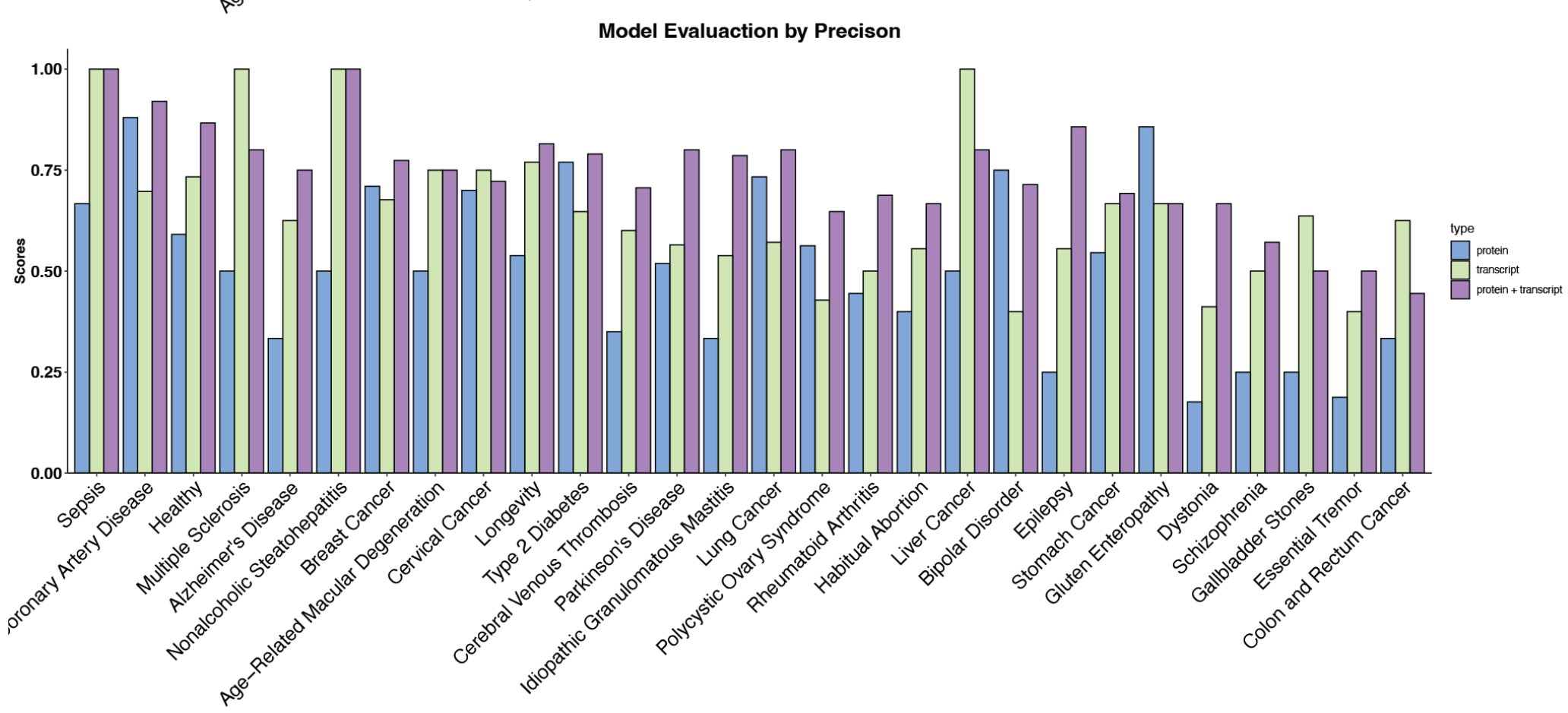
Model Evaluaction by Precison
Scores
0.00
0.25
0.50
0.75
1.00
type
protein
transcript
protein + transcript
Sepsis
oronary Artery Disease
Healthy
Multiple Sclerosis
Alzheimer's Disease
Nonalcoholic Steatohepatitis
Breast Cancer
Age−Related Macular Degeneration
Cervical Cancer
Longevity
Type 2 Diabetes
Cerebral Venous Thrombosis
Parkinson's Disease
Idiopathic Granulomatous Mastitis
Lung Cancer
Polycystic Ovary Syndrome
Rheumatoid Arthritis
Habitual Abortion
Liver Cancer
Bipolar Disorder
Epilepsy
Stomach Cancer
Gluten Enteropathy
Dystonia
Schizophrenia
Gallbladder Stones
Essential Tremor
Colon and Rectum Cancer

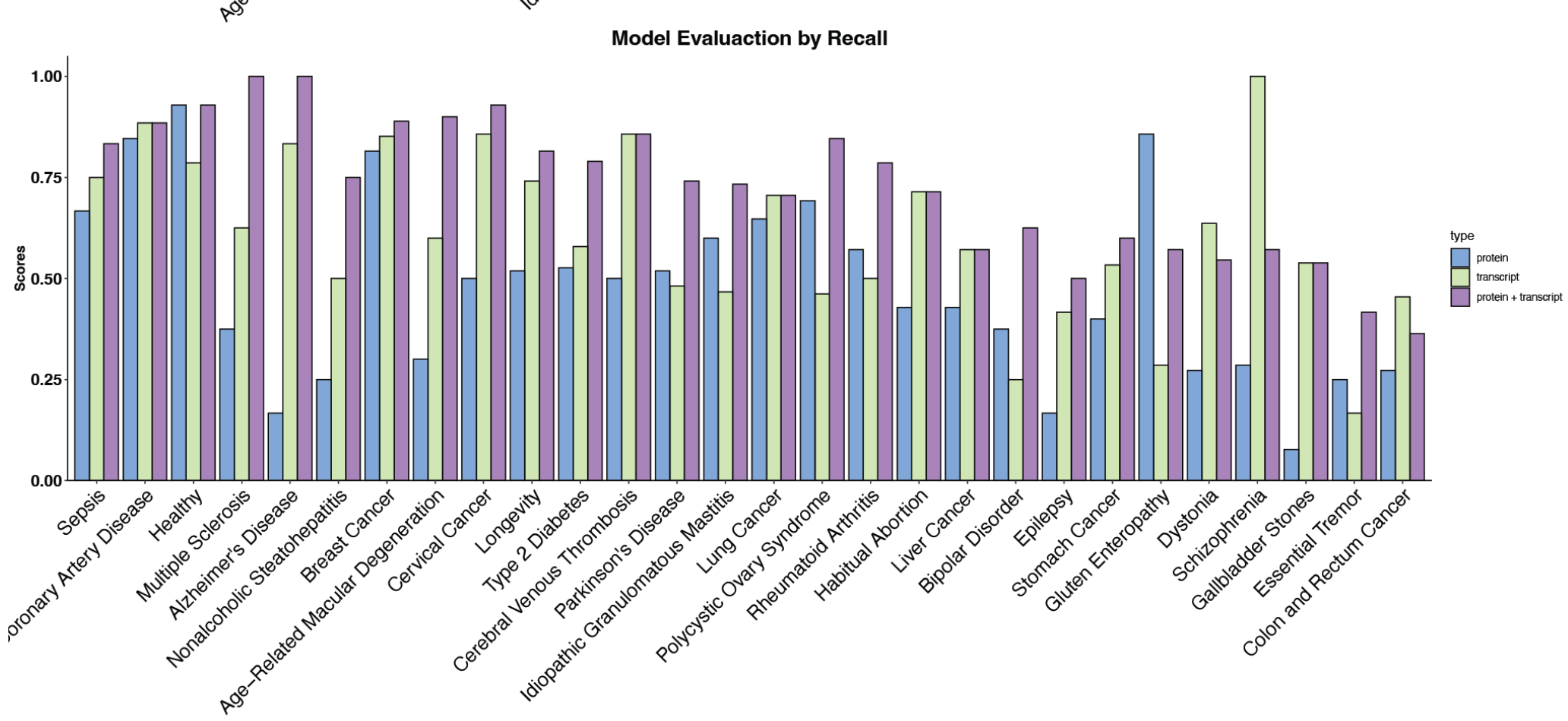
Model Evaluaction by Recall
Scores
0.00
0.25
0.50
0.75
1.00
type
protein
transcript
protein + transcript
Sepsis
oronary Artery Disease
Healthy
Multiple Sclerosis
Alzheimer's Disease
Nonalcoholic Steatohepatitis
Breast Cancer
Age−Related Macular Degeneration
Cervical Cancer
Longevity
Type 2 Diabetes
Cerebral Venous Thrombosis
Parkinson's Disease
Idiopathic Granulomatous Mastitis
Lung Cancer
Polycystic Ovary Syndrome
Rheumatoid Arthritis
Habitual Abortion
Liver Cancer
Bipolar Disorder
Epilepsy
Stomach Cancer
Gluten Enteropathy
Dystonia
Schizophrenia
Gallbladder Stones
Essential Tremor
Colon and Rectum Cancer

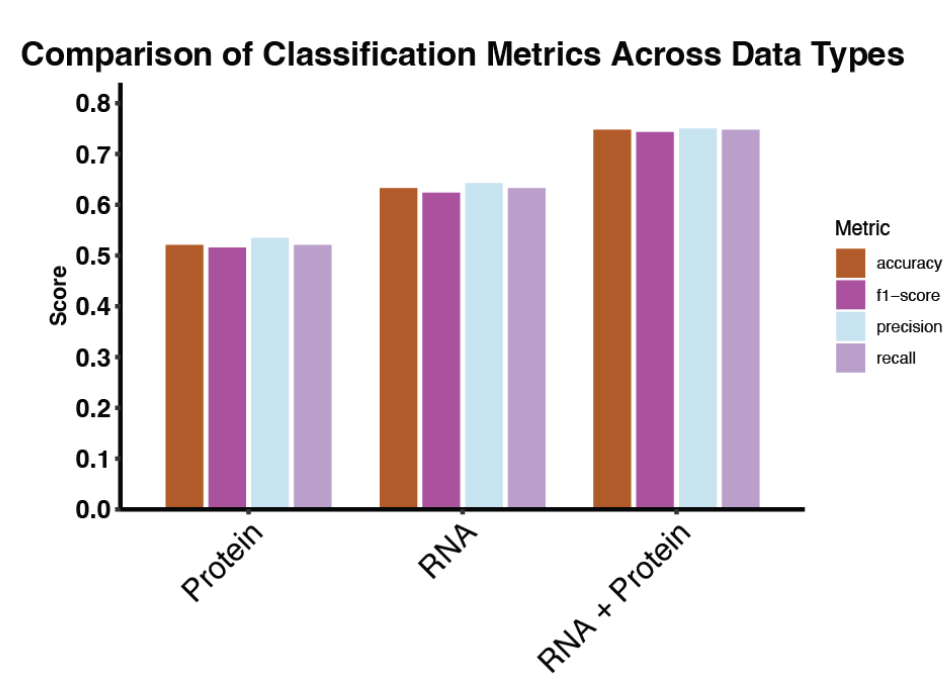
Comparison of Classification Metrics Across Data Types
Score
0.0
0.1
0.2
0.3
0.4
0.5
0.6
0.7
0.8
Protein
RNA
RNA + Protein
Metric
accuracy
f1−score
precision
recall

Supplementary Fig. 6

F1 scores, recall, and precision for RNA, protein, and combined RNA–protein data in a multi-class model distinguishing 28 health conditions using transcriptomic–proteomic–matched samples and a consistent training pipeline. The feature sets included the top 20 SHAP-ranked RNA features for the RNA model, the top 20 SHAP-ranked protein features for the protein model, and the combined top 10 RNA plus top 10 protein features for the integrated model. The lower panel shows the mean predictive performance across the three data types. Source data are provided in the Source data file.

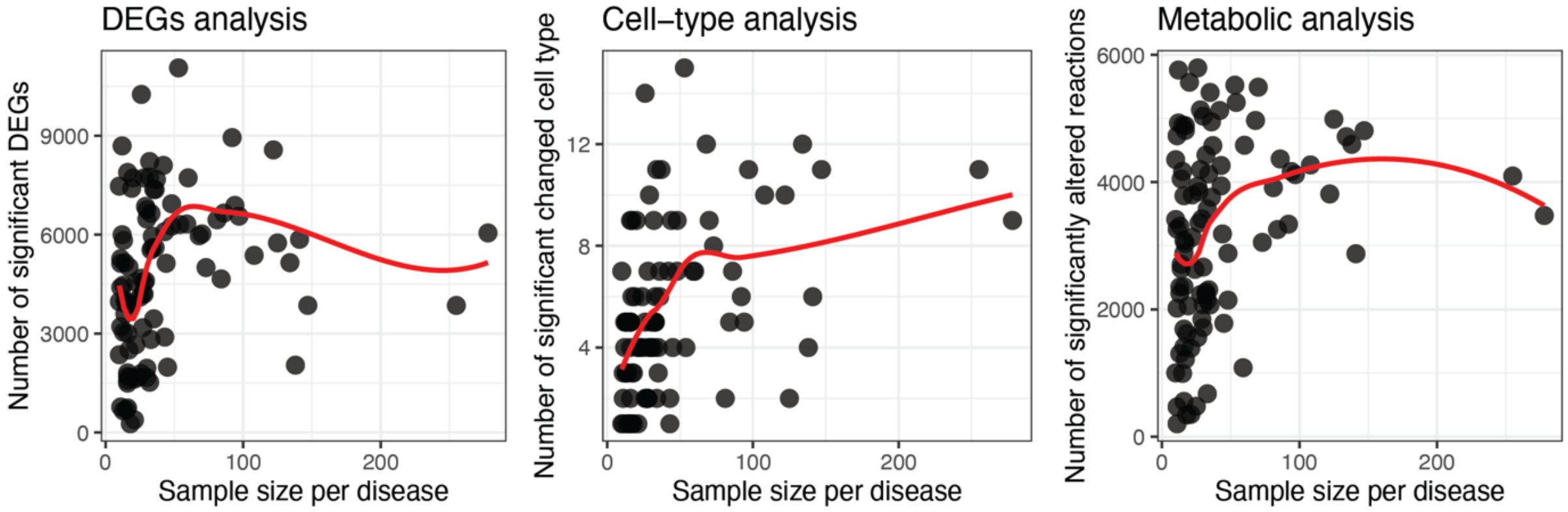

Supplementary Fig. 7
The relationship between sample size and the number of significant signals detected across differential expression analysis, CIBERSORTx cell-type deconvolution, and COMPASS metabolic activity estimation. No significant linear association was observed. Source data are provided in the Source data file.

Supplementary Table 1

Cohort Demographic Information

**Healthy Conditions**	**Size (WBT)**	**Size (Proteomics)**	**Male %**	**Age (Mean±SD)**
Healthy	238	46	147 (61.8%)	39.3 ± 16.8
Longevity	134	89	57 (42.5%)	84.6 ± 3.9
Rheumatoid Arthritis	94	47	22 (23.4%)	52.1 ± 14.6
Ankylosing Spondylitis	92	/	46 (50%)	41.2 ± 11.8
Atopic Dermatitis	68	/	49 (72.1%)	0.7 ± 1.5
Familial Mediterranean Fever	16	/	10 (62.5%)	18.6 ± 17.3
Systemic Lupus Erythematosus	16	/	1 (6.2%)	37.9 ± 10
Scleroderma	12	/	4 (33.3%)	47.1 ± 16.1
ANCA-Associated Vasculitis	11	/	5 (45.5%)	45.5 ± 15
Breast Cancer	108	89	0 (0%)	52.9 ± 14.5
Lung Cancer	97	58	91 (93.8%)	65.6 ± 9.8
Colon and Rectum Cancer	60	38	29 (48.3%)	59.5 ± 13.4
Stomach Cancer	59	49	39 (66.1%)	61.8 ± 11.9
Cervical Cancer	48	47	0 (0%)	53.4 ± 12.7
Esophageal Cancer	37	/	20 (54.1%)	59.8 ± 11.2
Musculoskeletal System Tumors	30	/	16 (53.3%)	36.3 ± 19
Bladder Cancer	29	/	28 (96.6%)	65.8 ± 11.5
Liver Cancer	28	23	14 (50%)	58.2 ± 12.9
Leukemia	16	/	9 (56.2%)	52.4 ± 11.3
Prostate Cancer	13	/	13 (100%)	70.8 ± 9.2

Bile Duct Cancer	11	/	5 (45.5%)	66 ± 9.8
Lymphoma	11	/	6 (54.5%)	46.2 ± 20
Pancreatic Cancer	10	/	6 (60%)	56 ± 17.1
Congenital Heart Diseases	255	/	153 (60%)	10.1 ± 4
Coronary Artery Disease	122	88	99 (81.1%)	62.7 ± 10
Mitral Valve Prolapse	84	/	22 (26.2%)	12 ± 3.9
Acute Rheumatic Fever	73	/	30 (41.1%)	13.5 ± 2.8
Cerebral Venous Thrombosis	70	46	33 (47.1%)	67.4 ± 18.2
Deep Vein Thrombosis	33	/	14 (42.4%)	46.4 ± 17.3
Pulmonary Embolism	32	/	13 (40.6%)	64.7 ± 15.3
Premature Patent Ductus Arteriosus	29	/	13 (44.8%)	0 ± 0.2
Hypertension	21	/	7 (33.3%)	61.7 ± 12.1
Arrhythmias	13	/	9 (69.2%)	11.2 ± 4.3
Aortic and Mitral Valve Stenosis and Insufficiency	11	/	6 (54.5%)	63.1 ± 9.2
Apical Dental Abscess	30	/	15 (50%)	26.9 ± 11.4
Periodontitis	26	/	8 (30.8%)	34.2 ± 12.7
Gallbladder Stones	86	42	38 (44.2%)	56 ± 15.9
Idiopathic Granulomatous Mastitis	54	/	0 (0%)	35.8 ± 7.7
Gluten Enteropathy	30	22	10 (33.3%)	37.1 ± 11.4
Colon Volvulus	20	/	15 (75%)	65.2 ± 17.2
Acute Pancreatitis	14	/	6 (42.9%)	56.4 ± 14.9
Type 2 Diabetes	147	63	66 (44.9%)	60.3 ± 11.6
Polycystic Ovary Syndrome	138	43	0 (0%)	25.8 ± 4.3
Childhood Hashimoto's Thyroiditis	48	/	6 (12.5%)	13.5 ± 3.3
Type 1 Diabetes	44	/	24 (54.5%)	9.9 ± 3.6
Diabetic Complications	22	/	14 (63.6%)	62.8 ± 9.3
Childhood Hypothyroidism	19	/	7 (36.8%)	8.1 ± 5.3
Precocious Puberty	17	/	0 (0%)	9.5 ± 3
Idiopathic Direct Hyperbilirubinemia	13	49	8 (61.5%)	0 ± 0
Sepsis	53	41	28 (52.8%)	71.5 ± 12.1
Hydatid Cyst	35	/	23 (65.7%)	29.8 ± 20
Chronic Hepatitis B	27	/	19 (70.4%)	45.4 ± 14.6
Acute Poststreptococcal Glomerulonephritis	18	/	11 (61.1%)	8.7 ± 4
Nonalcoholic Steatohepatitis	17	14	6 (35.3%)	51.5 ± 9.6
Brucellosis	16	/	11 (68.8%)	49.1 ± 17.4
Anaphylaxis	12	/	8 (66.7%)	38.7 ± 13.7
Tuberculosis	10	/	6 (60%)	44.3 ± 18.5
Gonarthrosis	35	/	8 (22.9%)	65.5 ± 7.8
Rotator Cuff Tear	14	/	3 (21.4%)	57.8 ± 10.3
Scoliosis	11	/	1 (9.1%)	14.8 ± 9
Parkinson's Disease	278	90	161 (57.9%)	62.8 ± 11.5
Epilepsy	125	41	55 (44%)	31.7 ± 11.3
Essential Tremor	81	40	43 (53.1%)	56 ± 16.4
Dystonia	43	38	19 (44.2%)	46.1 ± 13.7
Bipolar Disorder	34	25	20 (58.8%)	35 ± 13.2
Alzheimer's Disease	33	21	18 (54.5%)	74.3 ± 9.1
Schizophrenia	33	25	17 (51.5%)	43.1 ± 11.5
Multiple Sclerosis	32	26	6 (18.8%)	27.9 ± 9.3
Major Depressive Disorder	28	/	4 (14.3%)	38.6 ± 17.4

Atypical Parkinsonism	27	/	17 (63%)	63.7 ± 9.4
Frontotemporal Dementia	22	/	10 (45.5%)	69.2 ± 9.5
Blepharospasm	18	/	5 (27.8%)	59.7 ± 10.4
Febrile Convulsion	16	/	10 (62.5%)	2.2 ± 1.4
First-Episode Psychosis	15	/	6 (40%)	32.1 ± 12.9
Neurofibromatosis	15	/	5 (33.3%)	19.1 ± 13.8
Age-Related Macular Degeneration	45	33	23 (51.1%)	69.1 ± 9.1
Retinal Vein Occlusion	30	/	13 (43.3%)	64.3 ± 10.1
Glaucoma	25	/	17 (68%)	60.7 ± 15.5
Habitual Abortion	141	47	73 (51.8%)	30.7 ± 5.9
Drug Intoxication	36	/	12 (33.3%)	27.9 ± 10.1
Sudden Hearing Loss	21	/	11 (52.4%)	30.6 ± 25.6
Carbon Monoxide Poisoning	17	/	5 (29.4%)	43.8 ± 18.6
Non-Syndromic Congenital Anomaly	34	/	15 (44.1%)	0 ± 0
Childhood Isolated Growth Hormone Deficiency	26	/	12 (46.2%)	10.8 ± 3
Congenital Deafness	24	/	10 (41.7%)	4 ± 4.6
Childhood Renal Stones	16	/	12 (75%)	5.6 ± 5.7
Down Syndrome	14	/	10 (71.4%)	2.6 ± 4.1
Nocturnal Enuresis	13	/	6 (46.2%)	9.8 ± 1.8
Renal Stones	43	/	33 (76.7%)	49.7 ± 13.5
Vesicoureteral Reflux	36	/	16 (44.4%)	5 ± 3.8
Gynecomastia	32	/	32 (100%)	14 ± 1.9
Benign Prostatic Hyperplasia	16	/	16 (100%)	69.6 ± 7.7
Polycystic Kidney Disease	15	/	7 (46.7%)	48.7 ± 14
Pyelonephritis	10	/	4 (40%)	56.9 ± 23.4
Spontaneous Pneumothorax	42	/	39 (92.9%)	33.1 ± 18.3
Asthma	28	/	18 (64.3%)	16.4 ± 19.4
Chronic Obstructive Pulmonary Disease	26	/	16 (61.5%)	70.8 ± 11.1
Bronchopulmonary Dysplasia	17	/	11 (64.7%)	0 ± 0
**Total**	**4,444**	**1,280**	**2,164 (48.7%)**	**42.3 ± 25.1**
