# Supplementary material for: Whole blood transcriptomics atlas across human diseases
Source: Nat Commun. 2026 Oct 12;17:11547. doi: 10.1038/s41467-026-78440-w (PMC13748637; doi:10.1038/s41467-026-78440-w)
Supplement: Supplementary file 2 [file 41467_2026_78440_MOESM2_ESM.pdf]

nature portfolio

Peer Review File

Whole Blood Transcriptomics Atlas Across Human Diseases

Corresponding Author: Professor Adil Mardinoglu

This file contains all reviewer reports in order by version, followed by all author rebuttals in order by version.

Version 0:

Reviewer comments:

Reviewer #1

(Remarks to the Author)
The authors addressed most of my concerns in the revised manuscript and rebuttal letter.

However, the new analyses of related to cell composition – including CIBERSORTx and WCGNA – perhaps suggest the simple linear adjustment for cell composition are not sufficient for controlling confounding (eg the “we included CIBERSORTx-derived immune cell fractions as covariates in ANOVA models for the 500 most variable genes.” Section in the rebuttal). We had seen the same thing in the paper on DNA methylation as a proxy of cell composition and subsequent associations with age that I previously linked (https://link.springer.com/article/10.1186/gb-2014-15-2-r31 - Conclusions : “Note that due to the high levels of confounding we currently do not recommend regression approaches for adjustment purposes, but we note that RUV performed best for reducing the composition-based confounding”).

In both the CIBERSORTx and WCGNA sections, there are highly significant associations between cell composition and diagnosis. I therefore think it would be useful to extend the machine learning-based disease prediction analyses to include the resulting cell composition estimates per sample as one of the feature sets, and perform the following additional runs, evaluating the test set accuracy: a) Composition only, and b) Composition + RNA features? Basically I am wondering how Figure 7 looks if only cell composition estimates (and not RNA levels) are used as a feature set to classify patients by diagnosis, and how much additional information is provided by the RNA levels on top of composition.

Minor: the search bar on top of the browser at: https://www.phenomeatlas.org/wbt/ does not work

-Andrew Jaffe (please leave my signature in)

(Remarks on code availability)
The code is well documented and seems easy enough to follow (although I am admittedly not a strong python code developer). The github repository does not have access to all of the datasets so I did not try to run any of the code itself.

Reviewer #2

(Remarks to the Author)
I would like to thank the authors for the thorough revision. They clearly took the concerns raised in the initial review seriously, and the manuscript has improved substantially as a result.

The additional samples and diseases significantly increase the potential interest of such a paper, as a resource. The clarification of the machine learning framework is also appreciated. Finally, the use of independent datasets for testing the classifiers substantially strengthens the predictive claims compared to the original submission.

Overall, the study is now methodologically more solid and conceptually clearer.

However, there is still a major concern and a few minor points that I would like to raise.
My major concern is that:
1. The ethnicity covariate issue (described by all three reviewers) is not properly addressed by the authors in my opinion. Moreover, this part was not at all included in the manuscript. It triggers the following questions:

a. Why was the analysis done on only 40 samples (out of ~4000?), and how is this representative of the data?
b. How was the ethnicity assessment provided in the rebuttal letter computed exactly? There are no methods described. Were variants called from the transcriptomics data, or was there some additional genetics data available for these 40 patients? If the first option, why was it not done for the whole panel?

There are also a few minor points that I would strongly suggest addressing:

2. I think the primary value of this study lies in the resource it generates. However, the results are not currently disseminated in a practically useful manner. While the web portal is a welcome addition, it has limited utility in its present form. Direct access to the underlying data (e.g., gene lists, processed datasets, prediction models) would be substantially more valuable. In particular,
a. The authors state that raw data will be made available upon request. Although acceptable, I think that for such a resource it would be more valuable to deposit the raw data in a controlled-access repository such as dbGAP to ensure standardized, long-term availability.
b. The processed data and final models should also be made available through the Zenodo link provided, and, ideally, made directly accessible through the web portal. It may be on Zenodo already but as the data is embargoed its content cannot be evaluated (at least I don't have access to it).
c. The web portal requires an access token (which is shared for the reviewers). This raises concerns regarding long-term accessibility. The authors should clarify whether the portal will be fully open upon publication or will continue to require restricted access.

Furthermore,
3. the machine learning section would benefit from an even clearer and more explicit statement in the Methods describing how feature selection, model fitting, and validation were separated, simply to remove any remaining ambiguity.
4. in the discussion of cellular composition, it would be helpful to slightly tone down the interpretation and acknowledge that, even if cell fractions explain less variance globally, some disease-specific effects may still be partially mediated by compositional shifts.
5. for disorders with weaker replication, a short comment on why results may differ between cohorts (e.g. differences in patient characteristics, treatment, or sampling) would make the interpretation more balanced.

(Remarks on code availability)

Version 1:

Reviewer comments:

Reviewer #1

(Remarks to the Author)
The authors addressed my concerns around cell composition potentially driving the diagnosis predictions by assessing the three input features of RNA-only, cell count-only, and both, and showed that RNA features exclusively drove predictions.

While the RUV analyses were only included in the rebuttal and not the paper, that was technically not the correct way to run RUV - "control genes" should be selected from external data, and not features that do not associate with diagnosis within the target dataset. For example, in that prior epigenetics example I previously cited, "control probes" were selected to associate with cell type from a cell type-specific/sorted dataset and were not identified in bulk blood datasets of DNA methylation. This is the relevant portion of the discussion of the original RUV-2 paper:
"There may be a temptation to "discover" negative control genes. For example, a researcher may wish to find genes whose expression levels are not highly correlated with the factor of interest, label these genes as negative controls, and then adjust via RUV-2. The allure of this approach is clear—finding a set of negative controls would be much easier and could in fact be automated. However, we feel this approach is misguided. If there are unwanted factors that are correlated with the factor of interest, then the expression levels of the true negative controls should in fact be correlated with the factor of interest. Excluding genes correlated with the factor of interest would bias our estimate of the unwanted factors." [Gagnon-Bartsch & Speed, Biostatistics 2012].

(Remarks on code availability)
I have not re-reviewed the code since my last review.

Open Access This Peer Review File is licensed under a Creative Commons Attribution 4.0 International License, which permits use, sharing, adaptation, distribution and reproduction in any medium or format, as long as you give appropriate credit to the original author(s) and the source, provide a link to the Creative Commons license, and indicate if changes were made.
In cases where reviewers are anonymous, credit should be given to 'Anonymous Referee' and the source.
The images or other third party material in this Peer Review File are included in the article's Creative Commons license, unless indicated otherwise in a credit line to the material. If material is not included in the article's Creative Commons license and your intended use is not permitted by statutory regulation or exceeds the permitted use, you will need to obtain permission directly from the copyright holder.
To view a copy of this license, visit https://creativecommons.org/licenses/by/4.0/

RESPONSE TO REVIEWERS' COMMENTS

Reviewer #1 (Remarks to the Author):

The authors addressed most of my concerns in the revised manuscript and rebuttal letter.

However, the new analyses of related to cell composition – including CIBERSORTx and WCGNA – perhaps suggest the simple linear adjustment for cell composition are not sufficient for controlling confounding (eg the "we included CIBERSORTx-derived immune cell fractions as covariates in ANOVA models for the 500 most variable genes." Section in the rebuttal). We had seen the same thing in the paper on DNA methylation as a proxy of cell composition and subsequent associations with age that I previously linked (https://link.springer.com/article/10.1186/gb-2014-15-2-r31 - Conclusions : "Note that due to the high levels of confounding we currently do not recommend regression approaches for adjustment purposes, but we note that RUV performed best for reducing the composition-based confounding").

Response:

We thank the reviewer for this important and insightful suggestion. We carefully reviewed the referenced study and, following Jaffe & Irizarry (2014), implemented a Remove Unwanted Variation (RUV) approach using the RUVSeq framework as a more robust alternative to simple linear adjustment for cell composition.

Briefly, we identified 500 empirical negative control genes, defined as those with the highest adjusted P-values in an omnibus disease association test (i.e., least associated with any disease) and relatively stable expression (mean log2-TPM > 3). These genes were used to estimate latent factors via the RUVg method. We evaluated k = 1–3 factors and selected k = 1 (W1), as it showed the strongest and most interpretable correlation with inferred cell-type proportions. W1 was significantly correlated with CIBERSORTx-derived immune cell fractions, particularly neutrophils (Spearman r = 0.44) and CD8+ T cells (r = 0.43), indicating that this latent factor effectively captures variation related to cellular composition.

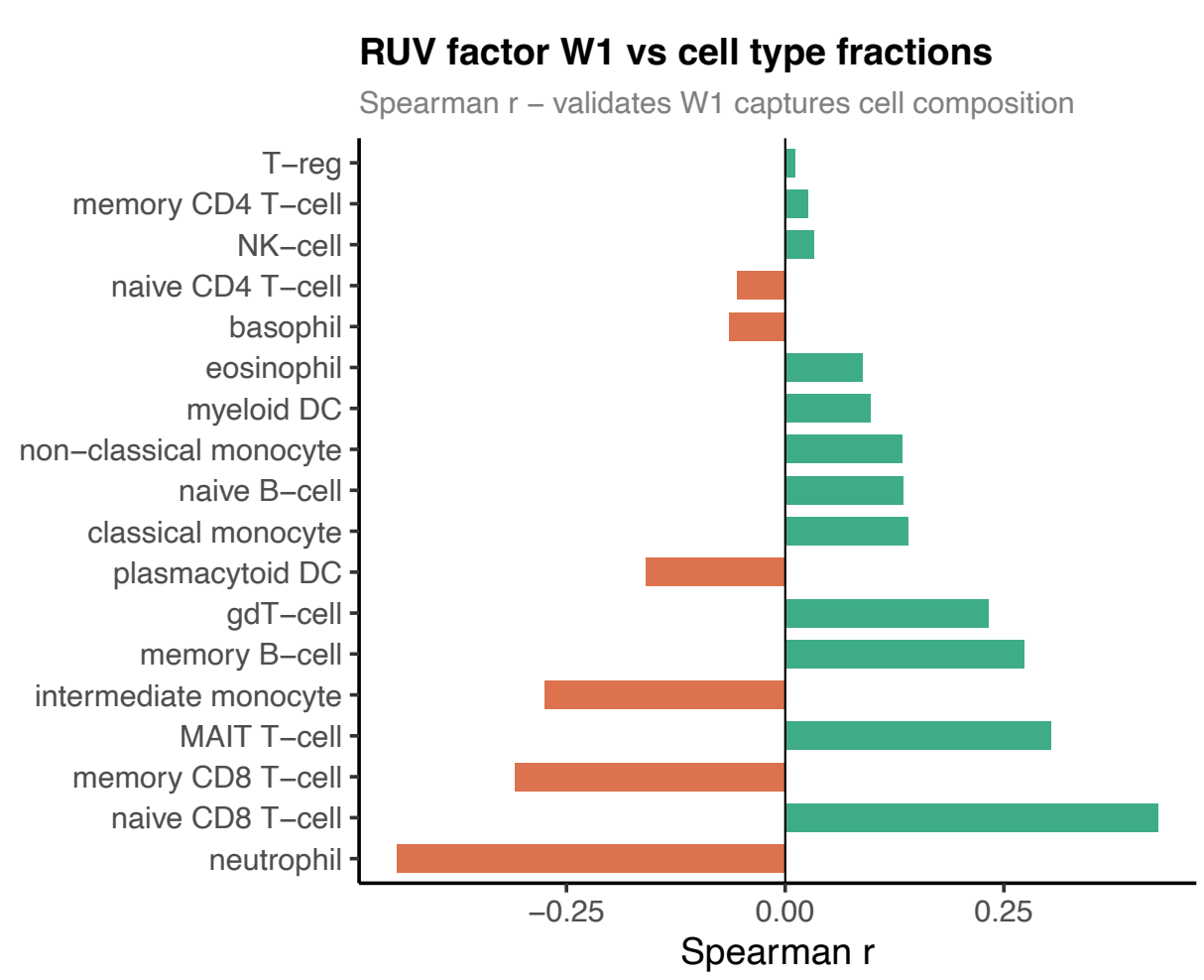

We then compared differential expression results across three models using limma for each disease vs. healthy comparison: (A) unadjusted, (B) linear cell fraction adjustment, and (C) RUV adjustment. Three models identified similar differentially expressed genes (DEGs). Effect sizes (log fold changes) of the DEGs were highly concordant between the unadjusted and RUV-adjusted models across all diseases (median Pearson r = 0.990, range 0.942–1.000), indicating that our primary findings are robust to compositional correction. These results support the conclusion that the majority of disease-associated transcriptional differences identified in our study reflect intrinsic gene regulatory changes rather than being driven solely by shifts in cellular composition.

Disease	DEGs number in ModelA	DEGs number in ModelB	DEGs number in ModelC	cor_AB	cor_AC
Breast Cancer	268	116	274	0.788	0.997
Congenital Heart Diseases	379	120	267	0.668	0.988
Coronary Artery Disease	826	795	731	0.914	0.990
Epilepsy	1434	116	509	0.702	0.942
Habitual Abortion	159	88	158	0.958	1.000
Longevity	2303	1692	1907	0.938	0.988
Parkinson's Disease	195	42	181	0.869	0.994
Polycystic Ovary Syndrome	174	89	179	0.953	1.000
Type 2 Diabetes	209	163	168	0.923	0.957

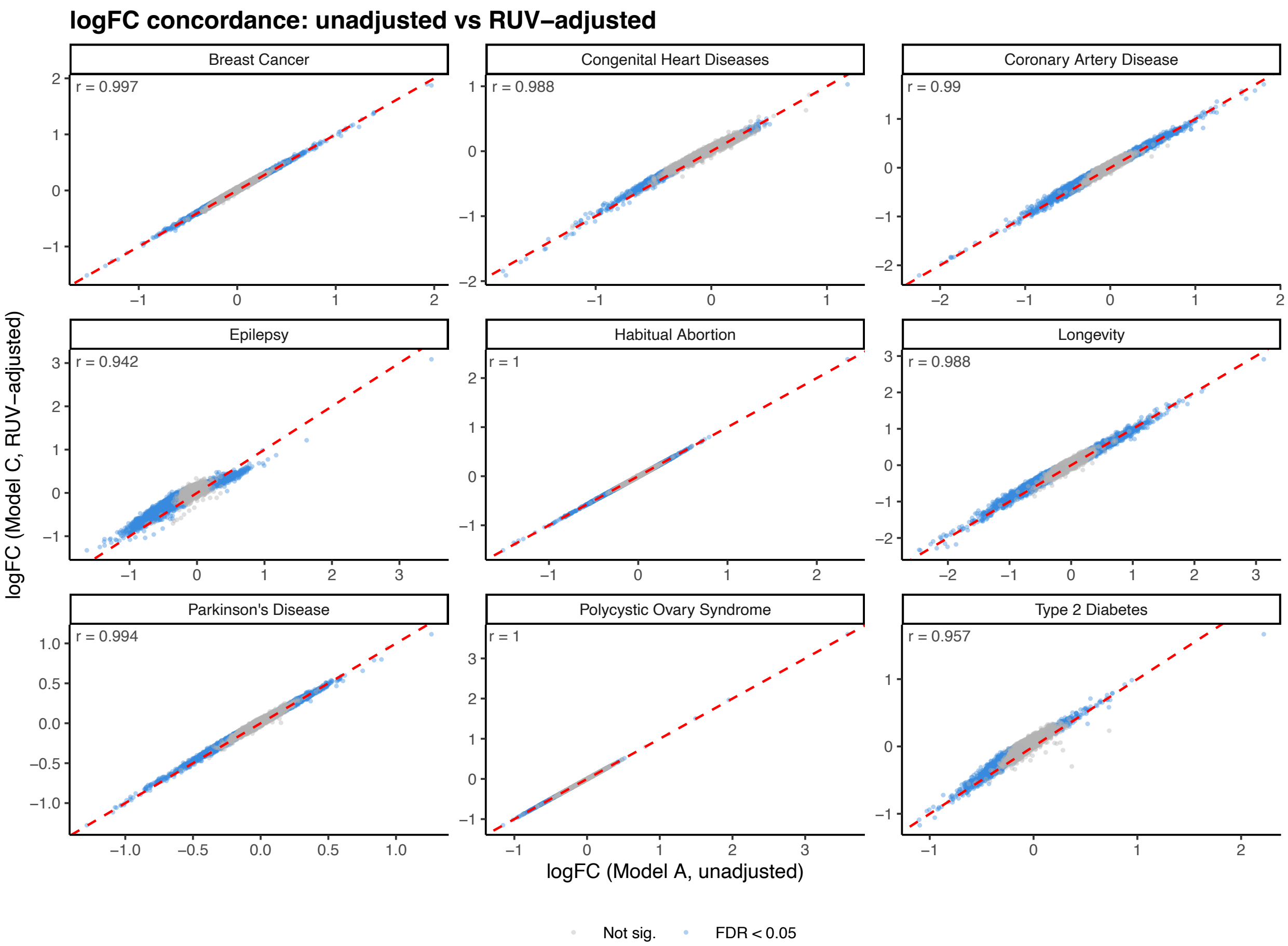

In both the CIBERSORTx and WCGNA sections, there are highly significant associations between cell composition and diagnosis. I therefore think it would be useful to extend the machine learning-based disease prediction analyses to include the resulting cell composition estimates per sample as one of the feature sets, and perform the following additional runs, evaluating the test set accuracy: a) Composition only, and b) Composition + RNA features? Basically I am wondering how Figure 7 looks if only cell composition estimates (and not RNA levels) are used as a feature set to classify patients by diagnosis, and how much additional information is provided by the RNA levels on top of composition.

Response:

To evaluate the contribution of different feature types, we compared three models following the same ML pipeline: RNA only (SHAP top10 genes), cell type proportions only, and a combination of both. As shown in Figure below, incorporating cell type proportions did not substantially improve predictive performance over the RNA-only model, suggesting that the selected gene features capture most of the signal. This indicates that cell type composition may play a limited role in disease classification, consistent with the notion that intrinsic

transcriptomic differences between diseases are primarily driven by gene expression patterns rather than shifts in cellular abundance.

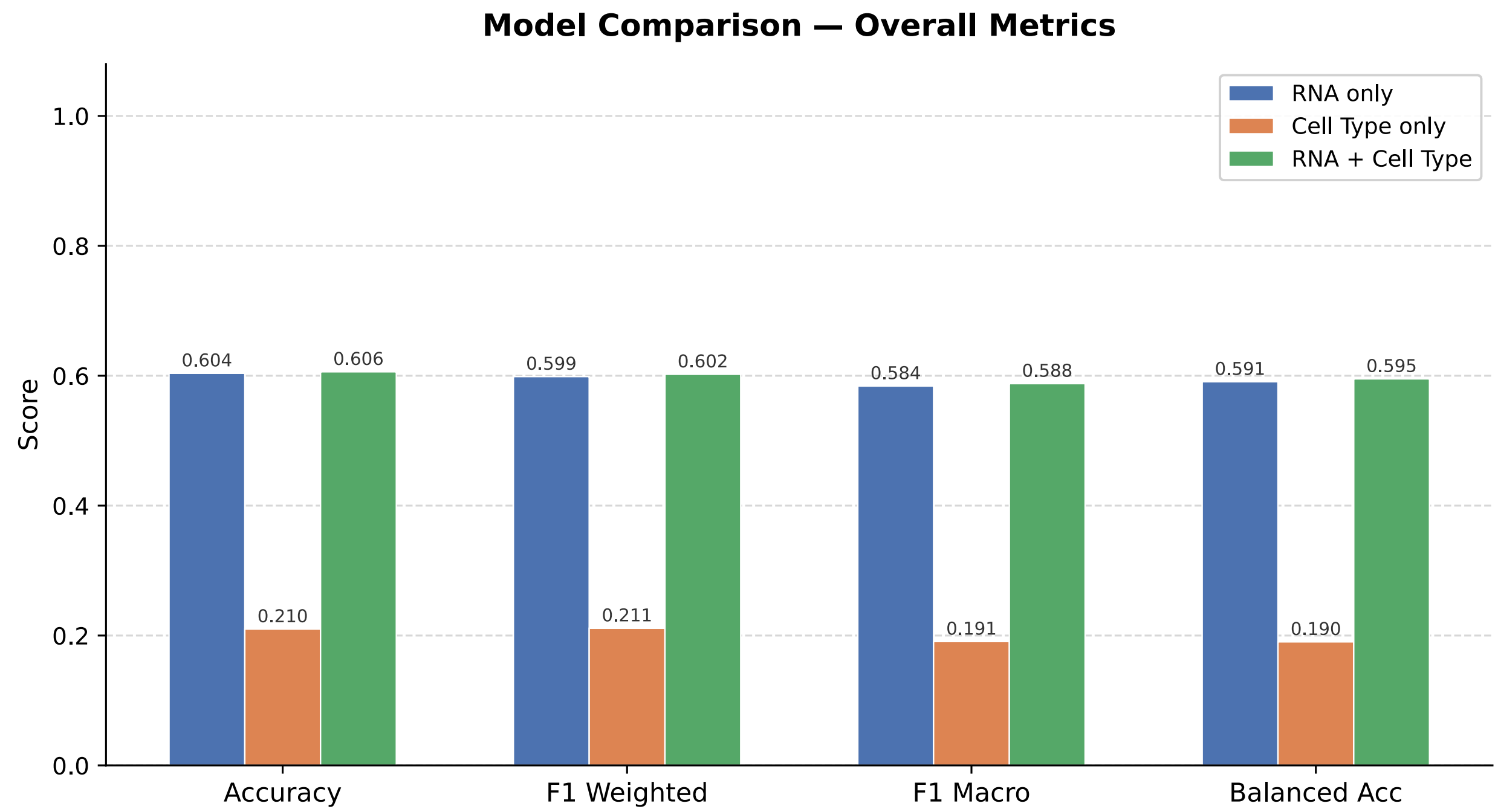

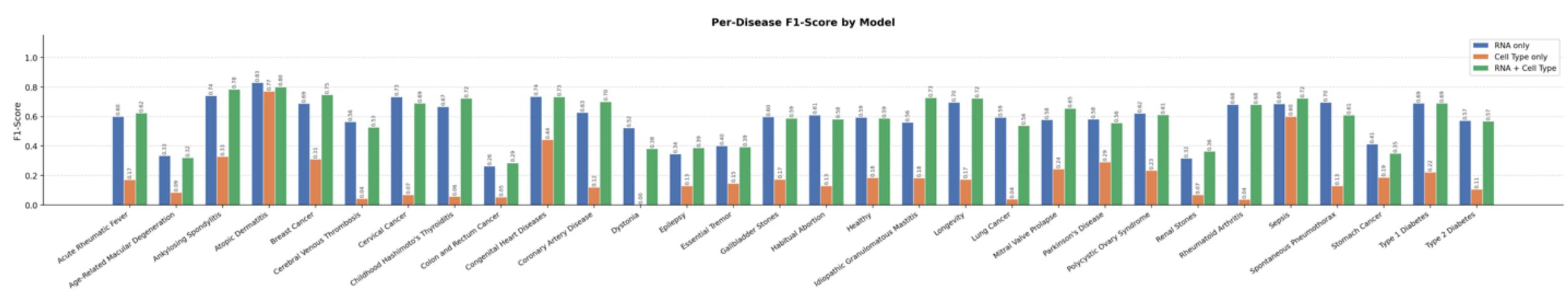

Minor: the search bar on top of the browser at: https://www.phenomeatlas.org/wbt/ does not work

-Andrew Jaffe (please leave my signature in)

We have identified the issue with the search bar and it has been resolved. We have also conducted a full review of the site to ensure all other sections are functioning as expected.

Reviewer #1 (Remarks on code availability):

The code is well documented and seems easy enough to follow (although I am admittedly not a strong python code developer). The github repository does not have access to all of the datasets so I did not try to run any of the code itself.

Thank you for your feedback. In addition, all relevant datasets are now publicly available on our website data section (https://www.phenomeatlas.org/wbt/explore) to ensure full reproducibility.

Reviewer #2 (Remarks to the Author):

I would like to thank the authors for the thorough revision. They clearly took the concerns raised in the initial review seriously, and the manuscript has improved substantially as a result.

The additional samples and diseases significantly increase the potential interest of such a paper, as a resource. The clarification of the machine learning framework is also appreciated. Finally, the use of independent datasets for testing the classifiers substantially strengthens the predictive claims compared to the original submission.

Overall, the study is now methodologically more solid and conceptually clearer.

However, there is still a major concern and a few minor points that I would like to raise. My major concern is that:
1. The ethnicity covariate issue (described by all three reviewers) is not properly addressed by the authors in my opinion. Moreover, this part was not at all included in the manuscript. It triggers the following questions:
a. Why was the analysis done on only 40 samples (out of ~4000?), and how is this representative of the data?
b. How was the ethnicity assessment provided in the rebuttal letter computed exactly? There are no methods described. Were variants called from the transcriptomics data, or was there some additional genetics data available for these 40 patients? If the first option, why was it not done for the whole panel?

Response:

We sincerely thank the reviewer for the encouraging feedback and apologize for the insufficient clarification in our previous response.

Regarding the ethnicity covariate issue, the ancestry analysis was performed using whole-genome sequencing (WGS) data, not derived from the transcriptomic data. We randomly selected 40 representative samples from the full cohort for this analysis. Ancestry inference was performed using ADMIXTURE, with the 1000 Genomes Project as the reference panel, which includes five super-populations (EAS, EUR, AFR, SAS, and AMR). We recognize that ideally the analysis would be performed across all ~4,000 samples. However, given the financial constraints of whole-genome sequencing at this scale, we consider the randomly selected 40 samples a reasonable preliminary assessment of the ethnic composition of our cohort.

There are also a few minor points that I would strongly suggest addressing:

2. I think the primary value of this study lies in the resource it generates. However, the results are not currently disseminated in a practically useful manner. While the web portal is a welcome addition, it has limited utility in its present form. Direct access to the

underlying data (e.g., gene lists, processed datasets, prediction models) would be substantially more valuable. In particular,
a. The authors state that raw data will be made available upon request. Although acceptable, I think that for such a resource it would be more valuable to deposit the raw data in a controlled-access repository such as dbGAP to ensure standardized, long-term availability.
b. The processed data and final models should also be made available through the Zenodo link provided, and, ideally, made directly accessible through the web portal. It may be on Zenodo already but as the data is embargoed its content cannot be evaluated (at least I don't have access to it).
c. The web portal requires an access token (which is shared for the reviewers). This raises concerns regarding long-term accessibility. The authors should clarify whether the portal will be fully open upon publication or will continue to require restricted access.

Response:

We thank the reviewer for these valuable suggestions regarding data dissemination and long-term accessibility. All processed data, gene lists, and final prediction models have now been made available through our website (https://www.phenomeatlas.org/wbt/explore). We apologize that, due to ethical regulations, all individuals working with the data must have prior approval. Therefore, we are unable to upload the data to an open-access repository at this stage. The data can be made available upon reasonable request by contacting the corresponding author.

The Zenodo repository is accessible to the reviewer via the token provided in the manuscript and will be fully and openly accessible upon publication.

The access token on the web portal was implemented during peer review. Upon publication, the portal will be made fully open and publicly accessible without any login or token requirement.

Furthermore,
3. the machine learning section would benefit from an even clearer and more explicit statement in the Methods describing how feature selection, model fitting, and validation were separated, simply to remove any remaining ambiguity.

We thank the reviewer for this suggestion. We have added an explicit statement to the Methods section to remove any remaining ambiguity (page 20).

"Data splitting was performed prior to all preprocessing steps. Feature selection, model selection, and hyperparameter tuning were applied exclusively to the training set. The test set was held out and used solely for final evaluation, ensuring that no information from the test set influenced any stage of feature selection or model fitting."

4. in the discussion of cellular composition, it would be helpful to slightly tone down the

interpretation and acknowledge that, even if cell fractions explain less variance globally, some disease-specific effects may still be partially mediated by compositional shifts.

We have revised the Discussion to provide a more balanced interpretation. Specifically, we have added the following statement on page 12

"An important consideration in WBT analysis is whether observed differences are driven by changes in cell type composition or by intrinsic gene expression within cells. Our results indicate that cell type composition explains only a limited proportion of the global variance, although a subset of the observed effects can still be partially attributed to compositional shifts."

5. for disorders with weaker replication, a short comment on why results may differ between cohorts (e.g. differences in patient characteristics, treatment, or sampling) would make the interpretation more balanced.

We have added the following statement on page 13.

"We also observed that certain diseases, such as gallbladder stones and dystonia, exhibited lower performance in the multi-omics models. This may be attributable to weaker systemic alterations detectable in WBT data, as well as relatively smaller sample sizes compared to other diseases."

RESPONSE TO REVIEWERS' COMMENTS

Reviewer #1 (Remarks to the Author):

The authors addressed my concerns around cell composition potentially driving the diagnosis predictions by assessing the three input features of RNA-only, cell count-only, and both, and showed that RNA features exclusively drove predictions.

While the RUV analyses were only included in the rebuttal and not the paper, that was technically not the correct way to run RUV - "control genes" should be selected from external data, and not features that do not associate with diagnosis within the target dataset. For example, in that prior epigenetics example I previously cited, "control probes" were selected to associate with cell type from a cell type-specific/sorted dataset and were not identified in bulk blood datasets of DNA methylation. This is the relevant portion of the discussion of the original RUV-2 paper:
"There may be a temptation to “discover” negative control genes. For example, a researcher may wish to find genes whose expression levels are not highly correlated with the factor of interest, label these genes as negative controls, and then adjust via RUV-2. The allure of this approach is clear—finding a set of negative controls would be much easier and could in fact be automated. However, we feel this approach is misguided. If there are unwanted factors that are correlated with the factor of interest, then the expression levels of the true negative controls should in fact be correlated with the factor of interest. Excluding genes correlated with the factor of interest would bias our estimate of the unwanted factors." [Gagnon-Bartsch & Speed, Biostatistics 2012].

Reviewer #1 (Remarks on code availability):

I have not re-reviewed the code since my last review.

Response:

We thank the reviewer for this important clarification regarding the correct implementation of RUV-2. We agree that negative control genes should be identified independently of the target dataset—for example, from external or cell type-specific reference data.

As the reviewer noted, the RUV analysis was presented only in the rebuttal as an exploratory, supplementary response to the reviewer's original question, and was not incorporated into the manuscript, nor used to support any of the reported results or conclusions. The evidence supporting our conclusions regarding cell composition is instead based on the three machine learning models trained on RNA-only, cell proportion-only, and combined features, which demonstrated that RNA features alone were the primary drivers of prediction performance.
