# Supplementary material for: Whole blood transcriptomics atlas across human diseases
Source: Nat Commun. 2026 Oct 12;17:11547. doi: 10.1038/s41467-026-78440-w (PMC13748637; doi:10.1038/s41467-026-78440-w)
Supplement: Supplementary file 3 [file 41467_2026_78440_MOESM3_ESM.pdf]

nature portfolio

Corresponding author(s): Adil Mardinoglu

Last updated by author(s): Jul 24, 2026

Reporting Summary

Nature Portfolio wishes to improve the reproducibility of the work that we publish. This form provides structure for consistency and transparency in reporting. For further information on Nature Portfolio policies, see our Editorial Policies and the Editorial Policy Checklist.

Statistics

For all statistical analyses, confirm that the following items are present in the figure legend, table legend, main text, or Methods section.

n/a	Confirmed	
☐	☒	The exact sample size (n) for each experimental group/condition, given as a discrete number and unit of measurement
☐	☒	A statement on whether measurements were taken from distinct samples or whether the same sample was measured repeatedly
☐	☒	The statistical test(s) used AND whether they are one- or two-sided<br>*Only common tests should be described solely by name; describe more complex techniques in the Methods section.*
☐	☒	A description of all covariates tested
☐	☒	A description of any assumptions or corrections, such as tests of normality and adjustment for multiple comparisons
☐	☒	A full description of the statistical parameters including central tendency (e.g. means) or other basic estimates (e.g. regression coefficient) AND variation (e.g. standard deviation) or associated estimates of uncertainty (e.g. confidence intervals)
☐	☒	For null hypothesis testing, the test statistic (e.g. F, t, r) with confidence intervals, effect sizes, degrees of freedom and P value noted<br>*Give P values as exact values whenever suitable.*
☒	☐	For Bayesian analysis, information on the choice of priors and Markov chain Monte Carlo settings
☐	☒	For hierarchical and complex designs, identification of the appropriate level for tests and full reporting of outcomes
☐	☒	Estimates of effect sizes (e.g. Cohen's d, Pearson's r), indicating how they were calculated

Our web collection on statistics for biologists contains articles on many of the points above.

Software and code

Policy information about availability of computer code

Data collection	Paired-end sequencing (200 cycles) was performed on the NovaSeq 6000 platform at SZA Omics Labs (Istanbul, Turkey). Raw BCL files were converted to FASTQ format and demultiplexed using DRAGEN (v4.2.7).
Data analysis	Data analysis was performed using R (version 4.3.1) and Python (version 3.11.4). Differential expression analysis of RNA-seq and proteomics data was conducted using the DESeq2 (v1.36.0) and limma (v3.58.1) R packages, respectively. Gene co-expression network analysis was performed using WGCNA (v1.73). Single-cell RNA-seq data were analyzed using Seurat (v5.2.1). Gene set enrichment analysis was conducted using clusterProfiler (v4.10.1).<br><br>Immune cell composition was estimated using CIBERSORTx (Newman et al., 2019) executed in command-line mode via Docker (cibersortx/fractions image). Flux balance analysis was performed using COMPASS (v0.9.10.2). TCR and BCR clonotypes were extracted from raw FASTQ files using MiXCR (v4.7.0). Machine learning analyses were performed using scikit-learn (v1.3.0).<br><br>All custom code for data analysis and visualization is available at: https://github.com/Mengzhensw/WBT_Atlas.

For manuscripts utilizing custom algorithms or software that are central to the research but not yet described in published literature, software must be made available to editors and reviewers. We strongly encourage code deposition in a community repository (e.g. GitHub). See the Nature Portfolio guidelines for submitting code & software for further information.

Data

Policy information about availability of data

All manuscripts must include a data availability statement. This statement should provide the following information, where applicable:

- Accession codes, unique identifiers, or web links for publicly available datasets
- A description of any restrictions on data availability
- For clinical datasets or third party data, please ensure that the statement adheres to our policy

Disease-level gene expression results are publicly available through https://www.phenomeatlas.org/wbt/. All other data supporting the findings of this study are provided within the paper, its Supplementary Information, and the Source Data. Owing to participant consent and legal restrictions, the individual-level datasets are not publicly available. These datasets may be made available for non-commercial research and validation purposes upon reasonable request to the corresponding author.

Research involving human participants, their data, or biological material

Policy information about studies with human participants or human data. See also policy information about sex, gender (identity/presentation), and sexual orientation and race, ethnicity and racism.

Reporting on sex and gender	In this study, the term "sex" refers to a biological attribute. Sex information was obtained from the original datasets (e.g., medical records) where available. no sex-stratified analyses were performed.
Reporting on race, ethnicity, or other socially relevant groupings	Race and ethnicity information were not explicitly analyzed in this study.
Population characteristics	The study population was derived from a patient cohort comprises individuals with 98 distinct health conditions.
Recruitment	The cohort was established by recruiting participants from January 2022 to September 2025 at Atatürk University Health Research and Application Center and Erzurum Regional Training and Research Hospital, Erzurum, Turkiye. Disease diagnoses were made according to clinical guidelines and the International Classification of Diseases, 10th Revision (ICD-10).
Ethics oversight	Ethics approval for all human samples and data collection was granted by the Ethical Committee for Clinical Research of the Medical Faculty at Atatürk University, Erzurum, Turkiye (Approval No: 45, dated 30.12.2021).

Note that full information on the approval of the study protocol must also be provided in the manuscript.

Field-specific reporting

Please select the one below that is the best fit for your research. If you are not sure, read the appropriate sections before making your selection.

- [x] Life sciences
- [ ] Behavioural & social sciences
- [ ] Ecological, evolutionary & environmental sciences

For a reference copy of the document with all sections, see nature.com/documents/nr-reporting-summary-flat.pdf

Life sciences study design

All studies must disclose on these points even when the disclosure is negative.

Sample size	No statistical methods were used to predetermine sample size. Sample sizes were determined by the number of participants recruited during the study period (January 2022 to September 2025) from Atatürk University Health Research and Application Center and Erzurum Regional Training and Research Hospital, representing a real-world patient cohort.
Data exclusions	Samples with missing data or failing quality control criteria were excluded prior to analysis. No additional exclusions were made.
Replication	Findings were validated across multiple independent cohorts and datasets where available, supporting the reproducibility of the results.
Randomization	Randomization was not applicable to this study. All analyses were conducted on observational datasets.
Blinding	Blinding was not applicable to this study, as no experimental interventions or group allocations were performed.

Reporting for specific materials, systems and methods

We require information from authors about some types of materials, experimental systems and methods used in many studies. Here, indicate whether each material, system or method listed is relevant to your study. If you are not sure if a list item applies to your research, read the appropriate section before selecting a response.

Materials & experimental systems

n/a	Involved in the study
☒	☐ Antibodies
☒	☐ Eukaryotic cell lines
☒	☐ Palaeontology and archaeology
☒	☐ Animals and other organisms
☒	☐ Clinical data
☒	☐ Dual use research of concern
☒	☐ Plants

Methods

n/a	Involved in the study
☒	☐ ChIP-seq
☒	☐ Flow cytometry
☒	☐ MRI-based neuroimaging

Plants

Seed stocks

Report on the source of all seed stocks or other plant material used. If applicable, state the seed stock centre and catalogue number. If plant specimens were collected from the field, describe the collection location, date and sampling procedures.

Novel plant genotypes

Describe the methods by which all novel plant genotypes were produced. This includes those generated by transgenic approaches, gene editing, chemical/radiation-based mutagenesis and hybridization. For transgenic lines, describe the transformation method, the number of independent lines analyzed and the generation upon which experiments were performed. For gene-edited lines, describe the editor used, the endogenous sequence targeted for editing, the targeting guide RNA sequence (if applicable) and how the editor was applied.

Authentication

Describe any authentication procedures for each seed stock used or novel genotype generated. Describe any experiments used to assess the effect of a mutation and, where applicable, how potential secondary effects (e.g. second site T-DNA insertions, mosiacism, off-target gene editing) were examined.
