# Supplementary material for: Whole blood transcriptomics atlas across human diseases
Source: Nat Commun. 2026 Oct 12;17:11547. doi: 10.1038/s41467-026-78440-w (PMC13748637; doi:10.1038/s41467-026-78440-w)
Supplement: Supplementary file 4 [file 41467_2026_78440_MOESM4_ESM.pdf]

Description of Additional Supplementary Files

Supplementary Data 1

Description: Differential expression genes list.

Supplementary Data 2

Description: Gene specificity list.

Supplementary Data 3

Description: WGCNA module.

Supplementary Data 4

Description: RNA-protein module.

Supplementary Data 5

Description: ML signatures.

Supplementary Data 6

Description: ML model and performance.
